# Cost-effectiveness of diagnostic and therapeutic interventions for chronic hepatitis C: a systematic review of model-based analyses

**DOI:** 10.1186/s12874-018-0515-9

**Published:** 2018-06-13

**Authors:** Rodolfo Castro, Louise Crathorne, Hugo Perazzo, Julio Silva, Chris Cooper, Jo Varley-Campbell, Daniel Savignon Marinho, Marcela Haasova, Valdilea G. Veloso, Rob Anderson, Chris Hyde

**Affiliations:** 1 Fundação Oswaldo Cruz, FIOCRUZ, Instituto Nacional de Infectologia Evandro Chagas, INI, Avenida Brasil, 4365, 21040-900, Manguinhos, Rio de Janeiro, Brazil; 2 Universidade Federal do Estado do Rio de Janeiro, UNIRIO, Instituto de Saúde Coletiva, Rio de Janeiro, Brazil; 3 University of Exeter Medical School, Evidence Synthesis & Modelling for Health Improvement, ESMI, Peninsula Technology Assessment Group, PenTAG, Exeter, UK; 4 Fundação Oswaldo Cruz, FIOCRUZ, Centro de Desenvolvimento Tecnológico em Saúde, CDTS, Rio de Janeiro, Brazil

**Keywords:** Hepatitis C, Cost-effectiveness analysis, Health technology assessment

## Abstract

**Background:**

Decisions about which subgroup of chronic hepatitis C (CHC) patients should be treated with direct acting anti-viral agents (DAAs) have economic importance due to high drug prices. Treat-all DAA strategies for CHC have gained acceptance despite high drug acquisition costs. However, there are also costs associated with the surveillance of CHC to determine a subgroup of patients with significant impairment. The aim of this systematic review was to describe the modelling methods used and summarise results in cost-effectiveness analyses (CEAs) of both CHC treatment with DAAs and surveillance of liver disease.

**Methods:**

Electronic databases including Embase and Medline were searched from inception to May 2015. Eligible studies included models predicting costs and/or outcomes for interventions, surveillance, or management of people with CHC. Narrative and quantitative synthesis were conducted. Quality appraisal was conducted using validated checklists. The review was conducted following principles published by NHS Centre for Research and Dissemination.

**Results:**

Forty-one CEAs met the eligibility criteria for the review; 37 evaluated an intervention and four evaluated surveillance strategies for targeting DAA treatment to those likely to gain most benefit. Included studies were of variable quality mostly due to reporting omissions. Of the 37 CEAs, eight models that enabled comparative analysis were fully appraised and synthesized. These models provided non-unique cost-effectiveness estimates in a specific DAA comparison in a specific population defined in terms of genotype, prior treatment status, and presence or absence of cirrhosis. Marked heterogeneity in cost-effectiveness estimates was observed despite this stratification. Approximately half of the estimates suggested that DAAs were cost-effective considering a threshold of US$30,000 and 73% with threshold of US$50,000. Two models evaluating surveillance strategies suggested that treating all CHC patients regardless of the staging of liver disease could be cost-effective.

**Conclusions:**

CEAs of CHC treatments need to better account for variability in their estimates. This analysis suggested that there are still circumstances where DAAs are not cost-effective. Surveillance in place of a treat-all strategy may still need to be considered as an option for deploying DAAs, particularly where acquisition cost is at the limit of affordability for a given health system.

**Electronic supplementary material:**

The online version of this article (10.1186/s12874-018-0515-9) contains supplementary material, which is available to authorized users.

## Background

Hepatitis C virus (HCV) was first described as non-A, non-B hepatitis in patients who presented with acute hepatitis after transfusion of blood products [1]. HCV is an enveloped RNA virus, which targets hepatocytes leading to liver damage [2]. Parenteral transmission due to intravenous drug use, followed by transfusion of blood products, before HCV screening, has been described as the most frequent routes of infection. However, HCV can also be transmitted sexually or vertically [3, 4]. Among patients exposed to HCV, a minority can spontaneously clear the virus, and around 66–82% of patients who still have detectable serum HCV RNA for six months should be considered as chronically infected (chronic hepatitis C [CHC]) [5].

Chronic Hepatitis C is a major global health burden, but it is treatable [6]. However, for economic reasons the treatment is still restricted or out of reach in several settings [7, 8]. New direct-acting antivirals (DAAs) are highly effective for HCV treatment [9] but are still relatively expensive in most countries. The decision about whether and what subgroup of CHC patients should be treated with DAAs has economic importance. Nevertheless, there are also costs associated with the surveillance management of CHC to determine the stage of liver disease for treating only a smaller group of patients with significant but reversible impairment. Recently, it was found that the surveillance of liver disease with transient elastography (TE) is an economically attractive alternative to liver biopsy [10]. All these options for monitoring and management of CHC make the decision process much more complex. Furthermore, treat-all DAA strategies for CHC have gained acceptance despite the high acquisition costs of DAA drugs in most countries [11]. However, the cost-effectiveness of these alternative strategies for deploying DAAs has not been examined in low and middle income countries possibly due to high budget impact.

Understanding the methods used in identified models and how they influence results is important. One important review was previously published [12] to describe and systematically review the methodological approaches in published cost-effectiveness analyses (CEA) of CHC treatment with DAAs. The current systematic review aims to extend the analysis to: (1) explore and discuss the variation in model characteristics, and (2) summarize the incremental cost-effectiveness ratios found in intervention studies for CHC, and surveillance of liver disease studies.

## Methods

The systematic review was made to answer the following research question:

What model structures and parameters have been used to estimate the cost-effectiveness or effectiveness of surveillance or treatment of people living with chronic hepatitis C and what are their conclusions?

The systematic review was carried out following the principles published by the National Health Service (NHS) Centre for Reviews and Dissemination [13].

### Eligibility criteria

Eligible studies included mathematical or simulation models predicting costs and/or outcomes applied for interventions, surveillance, or clinical management of people living with CHC. Ultimately, only comparative studies evaluating an intervention that included a DAA were eligible for inclusion in the review. Economic evaluations alongside clinical trials and isolated statistical models fitted to observed data were excluded.

Eligible studies included models used to evaluate DAAs as intervention compared with established treatment strategies. The comparator conditions for the including a model were limited to “no treatment” or regimens with pegylated interferon. Eligible surveillance studies were those which used biological markers, elasticity imaging techniques, or liver biopsy. Studies evaluating screening of blood donation to reduce exposure to HCV were not included.

### Search strategy

The search strategy was developed in conjunction with an experienced Information Specialist (CC) and is provided in Additional file 1.

The database searches were conducted from inception to May 2015. The following bibliographic resources were searched: MEDLINE, EMBASE, NHS EED (The Cochrane Library), HTA Library (The Cochrane Library) and LILACS were searched. No limits were used. Citation chasing was conducted on publications included in the review and the reference lists of identified systematic reviews were also scrutinized.

### Study selection, data extraction and quality assessment

Titles and abstracts were screened by nine researchers (RC, RA, HP, JVC, DM, MH, LC, JCALS, CH). Each pair of researchers were allocated ~ 600 titles/abstracts and screened for relevance against the inclusion criteria, disagreements were resolved by discussion. Papers selected for full text review were reviewed and screened by six researchers (RC, HP, JCALS, CH, JVC, LC).

Data extraction was carried out by six researchers (RC, HP, JCALS, CH, LC, JVC) using a template. Data were extracted from included studies by one researcher and checked by another.

The following aspects of the included studies’ methodology were reviewed: model type, HCV population, regimens, perspective, time horizon, discount rate, cycle length, and sponsor.

Studies were critically appraised using the Philips checklist for assessing the quality of model-based economic evaluations [14]. In line with the instructions accompanying the final checklist, where there was insufficient information available in the article to assess quality the item was marked ‘No’. Included studies were also quality assessed using the CHEERS checklist (for reporting quality) [15].

### Data synthesis

The results of included studies were analysed on the basis of visual inspection of the tabulated extracted data. When applicable, the mean and confidence interval of incremental cost-effectiveness ratios (ICERs) deflated to 2015 international dollar with purchasing power parity (PPP) were calculated. Quantitative data synthesis was conducted using the R environment [16].

### Changes to protocol

Prior to full-text screening the criteria for the review of intervention CEAs were revised to specify that only studies evaluating an intervention that included a DAA were eligible for inclusion in the review. Although data extraction was conducted for all eligible studies, only studies that enabled comparative analysis were critically appraised and included in the quantitative synthesis. This selection was made to focus on the studies which evaluated at least one DAA with another treatment protocol for CHC and reported results by Genotype.

## Results

**Fig. 1:**
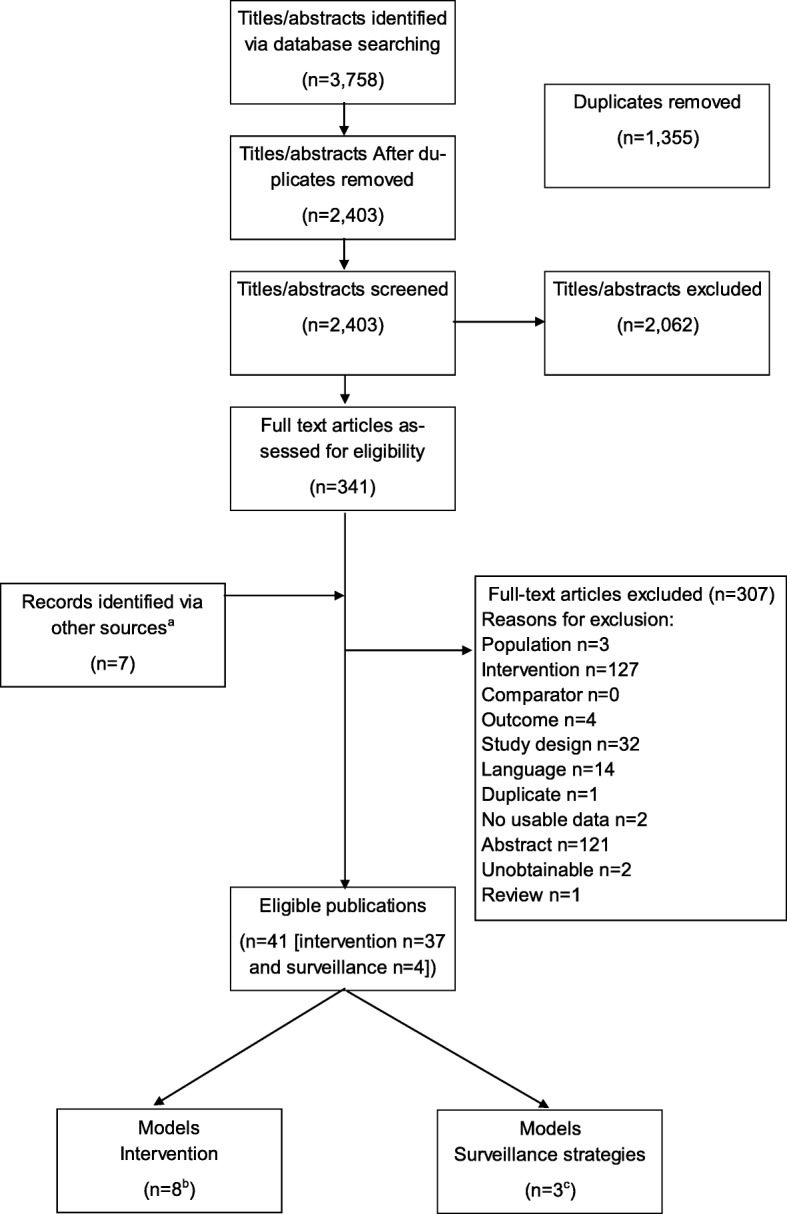
PRISMA flow diagram

The initial searches identified 2403 titles and abstracts after deduplication. Following screening 348 papers were requested for full-text review. Of these, seven further studies were identified when screening the reference lists of systematic reviews. A total of 307 publications were excluded at full text (see Additional file 2 for more detail). A total of 41 publications were eligible for inclusion in the review (see Tables 1 and 2): 37 publications [17–53] evaluated the cost-effectiveness of a DAA and four publications [54–57] reported three models evaluating the cost-effectiveness of surveillance methods. Of the 37 CEAs identified, eight were eligible for inclusion in the analysis [28, 29, 37, 39, 41, 44, 46, 47] as they evaluated at least one DAA with another treatment protocol for CHC. One study (Leleu et al., 2015) [35] did not report results by genotype and as such was not eligible for inclusion in the comparative analysis. The main countries in which the analyses were conducted were: United States of America (USA), United Kingdom (UK), Italy, Switzerland, and Spain. The study selection process is summarized in Fig. 1, Additional files 1 and 2.

**Table 1:** Summary characteristics of included models evaluating DAAs

First author Year Country	Model Type	HCV population	Regimens evaluated^a^	Perspective	Time horizon Discount rate Cycle length	Sponsor	Included in analysis, Y/N
Athanasakis 2015 Greece	Markov	Tx naïve & Tx experienced, G1	TT (BOC-PEG-RBV), DT (PEG-RBV)	3rd party payer	Lifetime 3%^a^ 1 wk	Merck Sharp & Dohme Corp.	N
Blazquez-Perez 2013 Spain	Markov	Tx naïve, G1	TT (BOC-PEG-RBV, TEL-PEG-RBV), DT (PEG-RBV)	Spanish NHS	Lifetime 3% 3 mths^a^	Unsupported^b^	N
Brogan 2014 USA	Markov	Tx naïve & Tx experienced, G1	TT (TEL-PEG-RBV), DT (PEG-RBV)	US payer perspective	Lifetime 3%^a^ 1 yr	Vertex Pharmaceuticals Incorporated	N
Camma 2013 Italy	Semi Markov	Tx experienced, G1, Aged 50+	TT (BOC/TEL-PEG-RBV), No Tx	Italian NHS	Lifetime 3%^a^ 1 yr	3P Solution	N
Camma 2012 Italy	Semi Markov	Tx naïve, G1, Aged 50+	TT (BOC/TEL-PEG-RBV), DT (PEG-RBV)	Italian NHS^c^	20-yr 3%^a^ 1 yr	3P Solution	N
Chan 2013 USA	Markov	Tx naïve, G1	TT (BOC-PEG-RBV, TEL-PEG-RBV), DT (PEG-RBV), No Tx	VHA Healthcare Organization	Lifetime 3%^a^ 1 yr	Dept. Of Veteran Affairs Health Services Research and DQERI	N
Chhatwal 2015 USA	Markov (Individual)	Tx naïve & Tx experienced, G1–4	TT (SOF-PEG-RBV, BOC-PEG-RBV, TEL-PEG-RBV), DT (SOF-LDV, SOF-RBV, PEG-RBV)	Third party payer	Lifetime 3%^a^ 1 wk	NIH (award #KL2TR000146)	N
Chhatwal 2013 USA	Markov	Tx experienced, G1	TT (BOC-PEG-RBV), DT (PEG-RBV)	Payer	Lifetime 3%^a^ 1 wk	Merck Sharp & Dohme Corp. (in part)	N
Cortesi 2015 USA	Semi Markov	Tx naïve, G1	TT (BOC-PEG-RBV, TEL-PEG-RBV)	Italian NHS	Lifetime 3%^a^ 1 yr	Unsupported	N
Cure 2015a Italy	Markov	Tx naïve & Tx experienced, G1–6	DT (PEG-RBV, SOF-RBV), TT (SOF-PEG-RBV, TEL-PEG-RBV, BOC-PEG-RBV), No Tx	Italian NHS	Lifetime 3%^a^ 3 mths & 1 yr^d^	Gilead Sciences	Y
Cure 2015b UK	Markov	Tx naïve & Tx experienced, G1–6	TT (SOF-PEG-RBV, BOC-PEG-RBV, TEL-PEG-RBV), DT (SOF-RBV, PEG-RBV), No Tx	UK NHS perspective	Lifetime 3.5%^a^ 3 mths & 1 yr^d^	Gilead Sciences	Y
Cure 2014a UK	Markov	Tx naïve, G1	TT (BOC-PEG-RBV, TEL-PEG-RBV)	UK NHS	Lifetime 3.5%^a^ 1 yr	Janssen Pharmaceuticals	N
Cure 2014b UK	Markov	Tx experienced, G1	TT (BOC-PEG-RBV, TEL-PEG-RBV)	Italian NHS	Lifetime 3.5%^a^ 1 yr	Janssen Pharmaceuticals	N
Dan 2015 Singapore	Markov	Tx naïve & Tx experienced, G1	TT (BOC-PEG-RBV), DT (PEG-RBV)	Public	Lifetime 3%^a^ 1 yr	Merck & Co Inc. & MSD Pharma (Singapore) Pte. Ltd.	N
Elbasha 2013 Portugal	Markov	Tx naïve & Tx experienced, G1	TT (BOC-PEG-RBV), DT (PEG-RBV)	Portuguese NHS	Lifetime 5%^a^ 1 wk	Merck Sharp & Dohme Corp.	N
Ferrante 2013 USA	Markov	Tx naïve, G1	TT (BOC-PEG-RBV), DT (PEG-RBV)	Payer	Lifetime 3%^a^ 1 wk	Schering Plough (part of Merck Sharp & Dohme Corp.)	N
Gimeno-Ballester 2016 Spain	Markov	Tx naïve, G1b	DT (SMV, DCV), TT (BOC-PEG-RBV, TEL-PEG-RBV)	Spanish NHS	Lifetime 3%^a^ 3 mths	Unsupported	N
Hagan 2014 USA	Markov	Tx naïve & Tx experienced	DT (SOF-SMV, SOF-RBV)	Societal	Lifetime 3%^a^ 1 yr	Grants from NIH and Department of Veterans Affairs	N
Leleu 2015 France	Markov	Tx naïve & Tx experienced, G1–4	TT (SOF-PEG-RBV, TEL-PEG-RBV), DT (PEG-RBV)	French NHS	Lifetime 2.5%^a^ 3 mths, 1 yr	Gilead Sciences	N [no usable data]
Linas 2015 USA	Monte Carlo Simulation	Tx naïve & Tx experienced, G2–3	TT (SOF-PEG-RBV), DT (SOF-RBV, PEG-RBV), No Tx	Payer	Lifetime 3%^a^ 1 mth	NIDA & NIAID	Y
Linas 2014 USA	Monte Carlo Simulation	HIV/HCV co-infected (Tx naïve, G1, non-cirrhotic)	TT (TEL-PEG-RBV), DT (PEG-RBV), No Tx	Health system	Lifetime 3%^a^ 1 mth	NIDA & NIAID	N
Liu 2014 USA	Markov	Tx naïve men, G1, Age 40+	TT (SOF-PEG-RV, BOC-PEG-RBV), DT (PEG-RBV), No Tx	Societal	Lifetime 3%^a^ 3 mths	US Dept. for Veteran Affairs, NIA, and NIH	Y
Liu 2012 USA	Markov	Tx naïve, G1	TT (BOC/TEL-PEG-RBV), DT (PEG-RBV)	Societal	Lifetime 3%^a^ 1 yr	Stanford Graduate Fellowship	N
McEwan 2014 Japan	Markov	Tx naïve & Tx experienced, G1b	TT (TEL-PEG-RBV) DT (DCV-ASV, PEG-RBV), No Tx	Japanese health system	Lifetime 2%^a^ 1 yr	Bristol-Myers Squibb	N
Najafzadeh 2015 USA	Discrete Event Simulation	Tx naïve, G1–3	TT (BOC-PEG-RBV, SOF-PEG-RBV, SOF-LED-RBV), DT (SOF-SIM, SOF-DCV, SOF-LED, SOF-RBV, PEG-RBV)	Societal	Lifetime 3%^a^ NA	CVS Health	Y
Petta 2014a Italy	Semi Markov	Tx naïve, G1, Age 50+	TT (SOF-PEG-RBV, BOC-PEG-RBV, TEL-PEG-RBV)	Italian National Health Service	Lifetime 3%^a^ 1 yr	3P Solution	N
Petta 2014b Italy	Semi Markov	Tx naïve, G1, Age 50+	TT (BOC-PEG-RBV), DT (PEG-RBV)	Italian National Health Service	Lifetime 3%^a^ 1 yr	Not reported	N
Pfeil 2015 Switzerland	Markov	Tx naïve & Tx experienced G1–4	TT (SOF-PEG-RBV, TEL-PEG-RBV, BOC-PEG-RBV), DT (PEG-RBV, SOF-RBV), No Tx	Swiss NHS	Lifetime 3%^a^ 1 yr	Gilead Switzerland	Y
Rein 2015 USA	Markov	Tx naïve, G1–4	DT (PEG-RBV, SOF-RBV, SIM-SOF), TT (SOF-PEG-RBV), No Tx	Healthcare	Lifetime 3%^a^ 1 yr	National Foundation for CDC & Prevention	N
Saab 2014 USA	Markov	Tx naïve, Tx experienced & Tx naïve with HIV coinfection	TT (SOF-PEG-RBV, BOC-PEG-RBV, TEL-PEG-RBV, SIM-PEG-RBV), DT (PEG-RBV)	3rd party payer	Lifetime 3%^a^ 1 yr	Gilead Sciences Inc.	Y
San Miguel 2014 Spain	Markov	Tx naïve & Tx experienced, G1–3	TT (SOF-PEG-RBV, BOC-PEG-RBV, TEL-PEG-RBV), DT (PEG-RBV, SOF-RBV)	Spanish NHS	Lifetime 3%^a^ 3 mths	Not reported^e^	Y
Tice 2015 USA	Markov	Tx naïve & Tx experienced, G1–3, Age 60+	TT (SOF-PEG-RBV, TEL-PEG-RBV, SMV-PEG-RBV, SOF-SMV-RBV), DT SOF-RBV), No Tx	US 3rd party payer	Lifetime 3%^a^ 1 yr	ICER & CTAF	N
Vellopoulou 2014 The Netherlands	Markov	Tx naïve & Tx experienced, G1	TT (SOF-PEG-RBV, BOC.PEG-RBV), DT (PEG-RBV)	Societal	Lifetime 4% costs; 1% outcomes 1 yr	Janssen-Cilag B.V.	N
Warren 2014 Australia	Markov	Tx naïve & Tx experienced, G1	TT (TEL-PEG-RBV), DT (PEG-RBV)		Lifetime 5%^a^ 1 yr	Janssen Australia Pty Ltd	N
Westerhout 2015 UK	Markov	Tx naïve & Tx experienced, G1, Age 50+	TT (SMV-PEG-RBV, TEL-PEG-RBV, BOC-PEG-RBV), DT (PEG-RBV)	UK NHS	Lifetime 3.5%^a^ 1 yr	Janssen EMEA	N
Younossi 2015 USA	Markov	Tx naïve & Tx experienced, G1	DT (SOF-LDV, SOF-SMV, SOF-RBV), TT (SOF-PEG-RBV, SMV-PEG-RBV, BOC-PEG-RBV), No Tx	US 3rd party payer	Lifetime 3%^a^ 1 yr	Gilead Sciences Inc.	N
Zhang 2015 USA	Markov (patient)	Tx naïve, G1–3	DT (PEG-RBV, SOF-RBV, LED-SOF, SIM-SOF), TT (SOF-PEG-RBV), ViekiraPak (OMB-PAR-RIT-DAS)	Unclear	Lifetime 3%^a^ 1 yr	National Science Foundation (grant #IIP-1361509 & #DGE1255832)	N

*Abbreviations*: *ASV* asunaprevir, *BOC* bocepravir, *BOC-PEG-RBV-48* fixed duration therapy for 48 weeks, *CDC* Center for disease control, *Corp.* Corporation, *DAA(s)* direct acting antiviral(s), *DAS* dasabuvir, *DCV* daclatasvir, *Dept.* department, *DQERI* development Quality Enhancement Research Initiative, *DT* dual therapy, *FD* fixed duration, *G* genotype, *IL* interleukin, *Inc.* incorporated, *LDV* ledipasvir, *mth(s)* month(s), *N* no, *NHS* National Health Service/system, *NIA* National Institute of aging, *NIAID* National Institute of Allergy and Infectious Diseases, *NIH* National Institutes of Health, *NIDA* National Institute on Drug Abuse, *OMB* ombitasavir, *PAR* paritaprevir, *PEG* pegylated interferon, *RBV* ribavarin, *RGT* response guided therapy, *RIT* ritonavir, *RVR* rapid virologic response, *SMV* simepravir, *SOF* sofosbuvir, *TEL* telaprevi[52]r, *TT* triple therapy, *Tx* treatment, *UK* United Kingdom, *US(A)* United States (of America), *VHA* veterans health association, *wk(s)* week(s), *Y* yes, *yr(s)* year(s)

Notes: ^a^Costs and health outcomes; ^b^No pharmaceutical company, government agency, or grant conducted as academic research; ^c^Unclear but study perspective limited to direct medical costs (Euros); ^d^The cycle length was 3 mths for Yr 1 and 2 and yearly thereafter; ^e^Assume unsupported no competing interests were reported but does not state explicitly. The cycle lengths were estimated using the data reported in each publication. A not applicable (NA) was added for studies without cycle length

Sources: Intervention models included in analysis: Cure 2015a [28], Cure 2015b [29], Linas, 2015 [37], Liu 2014 [39], Najafzadeh 2015 [41], San Miguel 2015 [47], Saab 2014 [46]; Intervention models excluded from analysis: Athanasakis 2015 [17], Blazques-Perez 2013 [18], Brogan 2014 [19], Camma 2012 [21], Camma 2013 [20], Chan 2013 [22], Chhatwal 2013 [23], Chhatwal 2015 [24], Cortesi 2015 [25], Cure 2014a [26], Cure 2014b [27], Dan 2015 [30], Elbasha 2013 [31], Ferrante 2013 [32], Gimeno-Ballester 2016 [33], Hagan 2014 [34], Leleu, 2015 [35]; Linas 2014 [36], Liu 2012 [38], McEwan 2014 [40], Petta 2014a [43], Petta 2014b [42], Rein 2015 [45], Tice 2015 [48], Vellopoulou 2014 [49], Warren 2014 [50], Westerhout 2015 [51], Younossi 2015 [52], Zhang 2015 [53]

**Table 2:** Summary characteristics of included models evaluating surveillance strategies

First author Year Country	Model Type	HCV population	Regimens evaluated^a^	Perspective	Time horizon Discount rate Cycle length	Sponsor
Canavan 2013 [56] UK	Markov	Newly diagnosed with chronic HCV and no fibrosis	• Intermittent biopsy followed by ultrasound and blood test every 6 mths • Annual biopsy followed by liver cancer screening at 6-mth intervals once cirrhosis identified • Replacing intermittent liver biopsy by TE with confirmation liver biopsy, followed by liver cancer screening at 6-month intervals once cirrhosis identified • Annual TE with confirmation liver biopsy, followed by liver cancer screening at 6-mth intervals once cirrhosis identified • Annual TE as a definitive test, followed by liver cancer screening at 6-mth intervals once cirrhosis identified • No surveillance of fibrosis stage	UK NHS (Hospital)	Lifetime 3.5%^a^ 3 mths	Lead author funded by MRC Population Health Science Fellowship
Crossan^b^ 2015 [54] UK	Markov	HBV, HCV (G1–4, with suspected fibrosis, who usually present for liver biopsy), ALD, NAFLD^c^	• TE • FibroTest • ARFI • Other invasive tests (including: DwMRI, • FibroIndex, contract-enhanced ultrasound, and Type IV collagen) • Liver biopsy	UK NHS	Lifetime 3.5%^a^ 3 mths	UK NIHR HTA Programme
Liu 2011 [57] USA	Markov	Tx naïve, G1–3	• FibroTest • FibroTest & liver biopsy • FibroTest rule-in • FibroTest rule-out • Liver biopsy only (recommended practice) • Immediate Tx	Payer	Lifetime 3%^a^ 6 mths	US NIH NIA Career development & Stanford Graduate Fellowship

*Abbreviations*: *ALD* alcoholic liver disease, *ARFI* acoustic radiation force impulse, *HBV* hepatitis B virus, *HCV* hepatitis C virus, *HTA* health technology assessment, *MRI* magnetic resonance imaging, *MRC* Medical Research Council, *mth(s)* month(s), *NAFLD* non-alcoholic fatty liver disease, *NHS* National Health Service/system, *NIA* National Institute of aging, *NIH* National Institutes of Health, *NIHR* National Institute for Health Research, *TE* transient elastography, *Tx* treatment, *UK* United Kingdom, *US* United States (of America

Notes: ^a^Costs and health outcomes; ^b^Model also reported in Tsochatzis et al., 2014 [55]; c Only the HCV population met the eligibility criteria for this review

Sources: Canavan 2013 [56], Crossan 2015 [54], Liu 2011 [57]; Tsochatzis 2014 [55]

## Model characteristics

### Therapeutic intervention

#### Model type and structure

A total of eight economic models evaluating the cost-effectiveness of interventions (including a DAA) for the treatment of chronic hepatitis C were included in the comparative analysis [28, 29, 37, 39, 41, 44, 46, 47]. Model characteristics are summarized in Table 1.

Most models were Markov-based, included the METAVIR stages and presence of cirrhosis as health states, with lifetime as time horizon and cycle length ranging from 30 days to 12 months. When the studies used Markov models with a previous decision tree, they were classified as Markov models. One study used a different approach for modelling with discrete event simulation [41]. The discount rates used ranged from 2.0 to 3.5% per year and the sensitivity analysis was performed by deterministic and/or probabilistic methods.

#### HCV population

Concerning characteristics of the population, studies frequently involved separate analysis for treatment naïve and experienced patients. Some of them evaluated the effects of the treatment for specific age groups. The most frequent HCV genotype was 1 but there were studies that included different range of genotypes from 1 to 6.

#### Perspective and sponsor

The most adopted perspectives were national health systems and third party payer, and some studies were developed with societal perspective. Regarding the funding, a considerable part was funded by pharmaceutical companies.

#### Regimens

A total of 126 different combinations of intervention comparison and population were described across 8 studies considering the following features: (i) Unique Combination of Intervention versus (vs) Comparator with Time in weeks of treatment duration (UCICT); (ii) HCV genotype; (iii) prior treatment status (naive versus treatment-experienced; and (iv) presence of cirrhosis (with versus without) (Table 3). In most comparisons, the population was treatment naive (*n* = 79 vs *n* = 47), 65 combinations were stratified by presence (*n* = 39) or absence (*n* = 26) of cirrhosis, and 61 combinations evaluated all patients (cirrhotic and non-cirrhotic) in the same group. Comparisons evaluating HCV Genotypes 1 and 3 (38.8 and 40.4%) were more prevalent in the studies compared to Genotype 2 (20.6%).

**Table 3:** Data available for analysis of all treatment comparisons used in intervention studies by population and study characteristics

		n	Genotype 1 n (%)	Genotype 2 n (%)	Genotype 3 n (%)
Prior treatment status	Treatment Experienced	47	15 (30.61)	9 (34.62)	23 (45.1)
	Treatment Naïve	79	34 (69.39)	17 (65.38)	28 (54.9)
Population	All Patients	61	27 (55.1)	13 (50)	21 (41.18)
	With Cirrhosis	39	14 (28.57)	8 (30.77)	17 (33.33)
	Without Cirrhosis	26	8 (16.33)	5 (19.23)	13 (25.49)
Articles	Linas, 2015	20	0 (0)	6 (23.08)	14 (27.45)
	Liu, 2014	1	1 (2.04)	0 (0)	0 (0)
	Najafzadeh, 2015	3	1 (2.04)	1 (3.85)	1 (1.96)
	Saab, 2014	16	16 (32.65)	0 (0)	0 (0)
	SanMiguel, 2014	8	2 (4.08)	2 (7.69)	4 (7.84)
	Cure, 2015 (Italy)	31	14 (28.57)	8 (30.77)	9 (17.65)
	Cure, 2015 (UK)	13	4 (8.16)	4 (15.38)	5 (9.8)
	Pfeil, 2015	34	11 (22.45)	5 (19.23)	18 (35.29)
Treatments	SOF PEG RBV 12 wk. vs BOC PEG RBV 48 wk	14	14 (28.57)	0 (0)	0 (0)
	SOF PEG RBV 12 wk. vs No Tx	7	4 (8.16)	0 (0)	3 (5.88)
	SOF PEG RBV 12 wk. vs PEG RBV 24 wk	11	0 (0)	2 (7.69)	9 (17.65)
	SOF PEG RBV 12 wk. vs PEG RBV 48 wk	19	13 (26.53)	0 (0)	6 (11.76)
	SOF PEG RBV 12 wk. vs TEL PEG RBV 48 wk	11	11 (22.45)	0 (0)	0 (0)
	SOF RBV 12 wk. vs No Tx	16	0 (0)	12 (46.15)	4 (7.84)
	SOF RBV 12 wk. vs PEG RBV 24 wk	10	0 (0)	8 (30.77)	2 (3.92)
	SOF RBV 12 wk. vs PEG RBV 48 wk	4	0 (0)	4 (15.38)	0 (0)
	SOF RBV 24 wk. vs No Tx	22	6 (12.24)	0 (0)	16 (31.37)
	SOF RBV 24 wk. vs PEG RBV 24 wk	7	0 (0)	0 (0)	7 (13.73)
	SOF RBV 24 wk. vs PEG RBV 48 wk	5	1 (2.04)	0 (0)	4 (7.84)
Total		126	49 (38.89)	26 (20.63)	51 (40.48)

Key: BOC = boceprevir; PEG = pegylated interferon; RBV = ribavirin; SOF = sofosbuvir; Tx = treatment; vs = versus; wk. = week

Based on data reported in included intervention models: Cure 2015a [28], Cure 2015b [29], Linas, 2015 [37], Liu 2014 [39], Najafzadeh 2015 [41], San Miguel 2015 [47], Saab 2014 [46]

Considering the comparative interventions in the included studies, the articles evaluated a total of 11 UCICTs. The UCICT “sofosbuvir (SOF) + ribavirin (RBV) 24 weeks (wks) versus (vs) No treatment (Tx)” was the most frequently evaluated in the included studies (*n* = 22; 6 for Genotype 1 and 16 for Genotype 3). The UCICT were specific in relation to genotypes which makes comparison encompassing more than one genotype difficult (Table 3).

### Surveillance

#### Model type and structure

A total of three economic models (reported in four publications) were identified evaluating surveillance strategies in chronic hepatitis C [54–57]. The models used for surveillance evaluation were similar to those used for DAA interventions, using a Markov modelling approach, one of them with a previous decision tree [54]. For one particular study, all patients started without fibrosis (METAVIR stage 0), further states were METAVIR stages 1–3 with separate stages constructed for diagnosed, undiagnosed, and misdiagnosed states, followed by Hepatocellular carcinoma (HCC), and radiofrequency thermal ablation [56]. Both other studies were based on METAVIR stages, with HCC, and Liver transplanted, and dead [57]; or all the previous states and an additional post-liver transplantation [54]. The time horizon used was lifetime for all studies. Model characteristics are summarised in Table 2.

#### HCV population

The HCV populations for surveillance studies were: newly diagnosed with chronic HCV and no fibrosis [56]; HBV, HCV genotypes 1–4, with suspected fibrosis, who usually present for liver biopsy [54]; and, treatment naïve, HCV genotypes 1–3 [57].

#### Perspective and sponsor

None of the surveillance studies were funded by industry [54–57]. The perspectives adopted were the National Health Systems [54, 56] or payer [57].

#### Regimens

Several alternatives were considered as surveillance regimens. The technologies used were: TE, FibroTest®, ARFI, DwMRI, FibroIndex, contract-enhanced ultrasound, and Type IV collagen, and liver biopsy. One of them included an immediate treatment as alternative [57].

### Quality appraisal

#### Therapeutic intervention

For the eight included intervention studies including DAAs [28, 29, 37, 39, 41, 44, 46, 47], the quality appraisal showed a considerable number of problems. Studies were quality assessed using both the Philips checklist [14] (see Table 4) and the CHEERS checklist [15] (see Table 5).

**Table 4:** Quality appraisal: Philips checklist (intervention and surveillance models)

	Intervention							Surveillance			
Philips criteria^a^	Cure, 2015a [28]	Cure, 2015b [28]	Linas, 2015 [37]	Liu, 2014 [39]	Najfzadeh, 2015	Pfeil, 2015 [44]	Saab, 2014 [46]	San Miguel, 2015 [47]	Canavan, 2013 [56]	Crossan, 2015^b^	Liu, 2011 [57]
S1	Unclear	Yes	Yes	Yes	Yes	Yes	Yes	Unclear	Yes	Yes	Yes
S2	Unclear	Yes	Yes	Yes	Yes	Yes	Yes	Yes	Yes	Yes	Yes
S3	Yes	Yes	Unclear	Yes	Yes	Yes	Yes	Yes	Yes	Yes	Yes
S4	No	Yes	Yes	Yes	Yes	Yes	Yes	Yes	Yes	Yes	Yes
S5	Unclear	Yes	Yes	Yes	Yes	Yes	Yes	Unclear	Yes	Yes	Yes
S6	Unclear	Yes	Unclear	Yes	Yes	Yes	Yes	Unclear	Yes	Yes	Yes
S7	Yes	Yes	Yes	Yes	Yes	Yes	Yes	Yes	Yes	Yes	Yes
S8	Yes	Yes	Yes	Yes	Yes	Yes	Yes	Yes	Yes	Yes	Yes
S9	Yes	Yes	Yes	Yes	Yes	Yes	Yes	Yes	Yes	Yes	Yes
D1	No	Unclear	Unclear	Yes	Unclear	Unclear	Unclear	No	Yes	Yes	Yes
D2	Unclear	Unclear	Unclear	No	Unclear	Unclear	No	No	Yes	Unclear	Yes
*D2A*	Yes	Yes	Unclear	Unclear	Yes	Yes	No	No	Yes	Yes	Yes
*D2B*	Unclear	Unclear	Unclear	Yes	Unclear	Unclear	Yes	No	Yes	Yes	Yes
*D2C*	Yes	Yes	Unclear	No	Yes	Yes	Unclear	No	Yes	Yes	Yes
D3	Unclear	No	Unclear	Yes	Yes	Yes	Yes	No	Yes	Yes	Yes
D4	Yes	No	No	Yes	Yes	Yes	No	No	Yes	Yes	Yes
*D4A*	Yes	No	Yes	Yes	Yes	Yes	Unclear	No	Yes	Yes	Yes
*D4B*	Yes	Unclear	Yes	Yes	Yes	Yes	Unclear	No	Yes	Yes	Yes
*D4C*	Yes	Yes	Yes	No	Yes	Yes	Yes	Yes	Yes	Yes	Yes
*D4D*	Yes	Yes	Unclear	Yes	Yes	Yes	Yes	Yes	Yes	Yes	Yes
C1	No	Yes	No	Yes	Yes	Yes	Unclear	No	Yes	Yes	Yes
C2	No	Yes	Yes	Yes	Yes	Yes	Yes	Yes	Yes	Yes	Yes

*Abbreviations*: *incl.* Including, *NA* not applicable, *QoL* quality of life, *Tx* treatment

Notes: Studies rated unclear due to reporting omissions (incl. Where detail not reported), ^a^Philips criteria detail, *S1* = Statement of decision problem/objective, *S2* = Statement of scope/objective, *S3* = Rationale for structure, *S4* = Structural assumptions, *S5* = Strategies/comparators, *S6* = Model Type, *S7* = Time horizon, *S8* = Disease states/pathways, *S9* = Cycle length, *D1* = Data identification, *D2* = Pre-model data analysis, *D2a* = Baseline data, *D2b* = Tx effect, *D2c* = QoL weights (utilities), *D3* = Data incorporation, *D4* = Assessment of uncertainty, *D4a* = Methodological, *D4b* = Structural, *D4c* Heterogeneity, *D4d* = Parameter, *C1* = Internal consistency, *C2* = External consistency, ^b^ Model also reported in Tsochatzis et al., 2014 [55]

Sources: Intervention models included in analysis: Cure 2015a [28], Cure 2015b [29], Linas, 2015 [37], Liu 2014 [39], Najafzadeh 2015 [41], San Miguel 2015 [47], Saab 2014 [46]. Surveillance models: Canavan 2013 [56], Crossan 2015 [54], Liu 2011 [57]; Tsochatzis 2014 [55]

**Table 5:** Quality appraisal: CHEERS checklist (intervention and surveillance models)

	Intervention								Surveillance		
CHEERS criteria^a^	Cure, 2015a [28]	Cure, 2015b [28]	Linas, 2015 [37]	Liu, 2014 [39]	Najfzadeh, 2015	Pfeil, 2015 [44]	Saab, 2014 [46]	San Miguel, 2015 [47]	Canavan, 2013 [56]	Crossan, 2015^b^ [54]	Liu, 2011 [57]
Q1	Yes	Yes	Yes	Yes	Yes	Yes	Yes	Yes	Yes	Yes	Yes
Q2	Unclear	Yes	Yes	Yes	Yes	Yes	Yes	Unclear	Yes	Yes	Yes
Q3	Yes	Yes	Yes	Yes	Yes	Yes	Yes	Yes	Yes	Yes	Yes
Q4	Yes	Yes	Yes	No	Yes	Yes	Yes	Yes	Yes	Yes	Yes
Q5	Yes	Yes	Unclear	Yes	Yes	Yes	Yes	Yes	Yes	Yes	Yes
Q6	Yes	Yes	Yes	Yes	Yes	Yes	Yes	Yes	Yes	Yes	Yes
Q7	Yes	Yes	Yes	Yes	Yes	Yes	Yes	Yes	Yes	Yes	Yes
Q8	Yes	Yes	Yes	Yes	Yes	Yes	Yes	Yes	Yes	Yes	Yes
Q9	Yes	Yes	Yes	Yes	Yes	Yes	Yes	Yes	Yes	Yes	Yes
Q10	Yes	Yes	Yes	Yes	Yes	Yes	Yes	Yes	Yes	Yes	Yes
Q11a	NA	NA	NA	NA	NA	NA	NA	NA	NA	NA	NA
Q11b	No	Unclear	Unclear	Yes	Unclear	Unclear	Unclear	Yes	Unclear	Yes	Unclear
Q12	Yes	Yes	Yes	Yes	Yes	Yes	Yes	Yes	Yes	Yes	Yes
Q13a	NA	NA	NA	NA	NA	NA	NA	NA	NA	NA	NA
Q13b	Yes	Yes	Yes	Yes	Yes	Yes	Yes	Yes	Yes	Yes	Yes
Q14	No	Yes	Yes	Yes	Yes	Yes	Yes	Unclear	Yes	Yes	Yes
Q15	Yes	Yes	Unclear	Unclear	Yes	Yes	Yes	Yes	Yes	Yes	Yes
Q16	Unclear	Unclear	Unclear	Yes	Yes	Unclear	Unclear	Unclear	Yes	Yes	Yes
Q17	Unclear	Unclear	Unclear	Unclear	Unclear	Unclear	Unclear	Unclear	Yes	Yes	Yes
Q18	No	Unclear	Yes	Unclear	Yes	Yes	No	Yes	Yes	Yes	Yes
Q19	No	Yes	Yes	No	Yes	Yes	Yes	Yes	Yes	Yes	Yes
Q20	NA	NA	NA	NA	NA	NA	NA	NA	NA	NA	NA
Q21	Yes	Yes	Yes	Yes	Yes	Yes	Yes	Yes	Yes	Yes	Yes
Q22	Yes	Yes	Yes	Yes	Yes	Yes	Yes	Yes	Yes	Yes	Yes
Q23	Yes	Yes	Yes	Yes	No	Yes	Unclear	Yes	Yes	Yes	Yes
Q24	Yes	Yes	Yes	Yes	Yes	Yes	Yes	Yes	Yes	Yes	Yes

*Abbreviations*: *incl*. Including, *NA* not applicable

Notes: Studies rated unclear due to reporting omissions (incl. Where detail not reported); ^a^CHEERS checklist criteria>#, *Q1* Identify the study as an economic evaluation, *Q2* Structured summary, *Q3* Provide an explicit statement of the broader context for the study, *Q4* Base-case population and subgroups analyzed, *Q5* State relevant aspects of the system(s) in which the decision(s) need(s) to be made, *Q6* Study perspective, *Q7* Interventions or strategies being compared, *Q8* Time horizon(s), *Q9* Discount rate(s) for costs and outcomes, *Q10* Outcomes measured, *Q11a* Single study–based estimate(s), *Q11b* Synthesis-based estimate(s), *Q12* Population and methods used to elicit preferences for outcomes, *Q13a* Single study–based economic evaluation: estimation of resource use, *Q13b*, Model-based economic evaluation: estimation of resource use, *Q14* Dates of the estimated resource quantities and unit costs, *Q15* Type of decision-analytic model used, *Q16* Structural or other assumptions underpinning the decision-analytic model, *Q17*, Analytic methods supporting the evaluation, *Q18* Report the values, ranges, references, and if used, probability distributions for all parameters, *Q19* Mean values for the main categories of estimated costs and outcomes of interest, as well as mean differences between the comparator groups (ICER where applicable), *Q20*, Single study–based economic evaluation: Effects of sampling uncertainty for estimated incremental cost, incremental effectiveness, and incremental cost-effectiveness, together with the impact of methodological assumptions (such as discount rate, study perspective), *Q21* Report differences in results that can be explained by variations between subgroups, *Q22* Summarize key study findings and describe how they support the conclusions reached. Limitations and generalizability of the findings; Q23, Source of funding; Q24, Conflict of interest; ^b^Model also reported in Tsochatzis et al., 2014 [55]

Sources: Intervention models included in analysis: Cure 2015a [28], Cure 2015b [29], Linas, 2015 [37], Liu 2014 [39], Najafzadeh 2015 [41], San Miguel 2015 [47], Saab 2014 [46]. Surveillance models: Canavan 2013 [56], Crossan 2015 [54], Liu 2011 [57]; Tsochatzis 2014 [55]

Considering the Philips checklist [14], only three of the items (“S7 - Time horizon”; “S8 - Disease states/pathways”; “S9 - Cycle length”) were fully accomplished by all included studies. The items which showed a higher frequency of problems were: “D1 - Data identification”; “D2 - Pre-model data analysis”; “D3 - Data incorporation”; “D4 - Assessment of uncertainty”; and, “C1- Internal consistency”. In summary, all [28, 29, 37, 41, 44, 46, 47] but one [39] of the intervention studies did not describe sufficiently or did not use systematic reviews to estimate parameters; all eight included studies were rated as “Unclear” or “No” for pre-model analysis [28, 29, 37, 39, 41, 44, 46, 47]; four studies were rated unclear or did not provide distributions for data incorporated [28, 29, 37, 47]; and, half of the studies failed in terms of assessment of uncertainty [29, 37, 46, 47].

Using the CHEERS checklist [15], slightly improved results in terms of study reporting quality were found, particularly when considering the number of checklist criteria for which problems were identified. However, the included studies failed to meet acceptable criteria for the following four questions: “Q11b - Synthesis-based estimates”; “Q16 - Describe all structural or other assumptions underpinning the decision-analytic model”; “Q17 - Describe all analytic methods supporting the evaluation”, and “Q18 - Report the values, ranges, references, and if used, probability distributions for all parameters”. The biggest problems identified in the CHEERS evaluation were: only two studies [39, 47] did not fail and five studies [29, 37, 41, 44, 46] were rated “Unclear” for the use of synthesis-based estimates; only two of the eight included studies described structural or other assumptions underpinning the decision models [39, 41]; all the studies had unclear description of analytic methods that supported the evaluations [28, 29, 37, 39, 41, 44, 46, 47]; and, four studies had important issues, such as, not reporting probabilities, range of estimates, or not reporting sufficient detail the sensitivity analysis [28, 29, 39, 46]. One aspect that was positive was that all the studies employed sensitivity analysis.

Considering the quality of the parameters used for calculation of ICERs, QALYs and costs, in some studies did not have sufficient description regarding uncertainty (Table 5, Q18). Among the papers that evaluated DAAs, the source of utility parameters was scientific literature, likewise for the transition probability parameters. However, the parameterization of costs occurred exclusively with data from scientific literature in four papers [37, 39, 41, 46]. The same number of the studies [28, 29, 44, 47] used cost data from the payer, mostly from the local health systems. It is also worth noting the use of expert opinion for the parameterization of costs. This approach was used by two studies [28, 29].

Hence, structural or other assumptions, analytic methods supporting the evaluation, and even justification, validation and calibration of the decision-analytic model were points unclear for a considerable part of the included studies. Regarding the calibration or validation, four studies [28, 29, 44, 46], reported the validation of the model structure. Three studies [41, 44, 47] reported the validation of the outputs of the models. One study [46] also reports the validation of inputs.

#### Surveillance

Four publications reported decision analytic models evaluating surveillance strategies [54–57]; however, two publications reported the same model and were quality appraised as one [54, 55]. Studies were also quality assessed using both the Philips checklist (see Table 4) and the CHEERS checklist (see Table 5).

Using the Philips checklist [14], the results for surveillance studies were very positive with just one study [54] evaluated as “Unclear” for Question D2, due to reporting omissions for pre-model data analysis (Table 4). The same pattern was observed with CHEERS (Table 5), whose most of the items of this checklist were accomplished by the three included surveillance studies. The exception was Question Q11b in which two studies [54, 57] were evaluated as “Unclear” in terms of description of the methods used to identify included studies and synthetize clinical effectiveness data.

### Synthesis results

#### Therapeutic intervention

In summary, 62 different and not dominated comparisons were described in the 8 included studies (*n* = 9 combinations had negative ICERs) for each UCICT for the same patient profile stratified by genotype, treatment naive or experienced and presence of cirrhosis). A total of 29 comparisons were evaluated only once in the eight included papers. In addition, the UCICTs “SOF + pegylated interferon (PEG) + ribavirin (RBV) 12 wks vs boceprevir (BOC) PEG RBV 48 wks” and “SOF PEG RBV 12 weeks vs PEG RBV 48 wks”, were the most frequent described (*n* = 5 studies). For those, the mean and 95% confidence interval (CI) of ICER in international dollar PPP of 2015 were calculated (Table 6).

**Table 6:** Synthesis of ICERs from the included intervention studies when available with more than one comparison

Genotype	Response	Population	Treatment	n	Mean	95% CI
1	Tx Naïve	All Patients	SOF PEG RBV 12 wk. vs BOC PEG RBV 48 wk	5	$18,499.62	6871.38; 30,127.86
1	Tx Naïve	All Patients	SOF PEG RBV 12 wk. vs PEG RBV 48 wk	5	$26,460.59	18,342.69; 34,578.49
2	Tx Naïve	All Patients	SOF RBV 12 wk. vs PEG RBV 24 wk	5	$88,099.60	68,301.59; 107,897.61
3	Tx Naïve	All Patients	SOF PEG RBV 12 wk. vs PEG RBV 24 wk	4	$41,080.16	28,962.61; 53,197.71
3	Tx Experienced	All Patients	SOF PEG RBV 12 wk. vs PEG RBV 48 wk	4	$29,092.09	9276.64; 48,907.54
1	Tx Naïve	All Patients	SOF PEG RBV 12 wk. vs TEL PEG RBV 48 wk	3	$25,954.71	− 242.37; 52,151.79
1	Tx Naïve	All Patients	SOF RBV 24 wk. vs No Tx	3	$61,607.34	43,706.46; 79,508.22
2	Tx Naïve	All Patients	SOF RBV 12 wk. vs No Tx	3	$13,411.94	6648.19; 20,175.69
2	Tx Experienced	All Patients	SOF RBV 12 wk. vs PEG RBV 48 wk	3	$38,521.07	8866.40; 68,175.74
3	Tx Naïve	With Cirrhosis	SOF PEG RBV 12 wk. vs PEG RBV 24 wk	3	$15,496.88	5997.08; 24,996.68
3	Tx Experienced	All Patients	SOF RBV 24 wk. vs No Tx	3	$34,349.09	23,903.12; 44,795.06
3	Tx Experienced	With Cirrhosis	SOF RBV 24 wk. vs No Tx	3	$61,199.47	−11,419.99; 133,818.93
3	Tx Naïve	All Patients	SOF RBV 24 wk. vs No Tx	3	$26,708.10	20,272.80; 33,143.40
3	Tx Naïve	With Cirrhosis	SOF RBV 24 wk. vs No Tx	3	$16,688.78	5501.36; 27,876.20
1	Tx Experienced	With Cirrhosis	SOF PEG RBV 12 wk. vs BOC PEG RBV 48 wk	2	$8155.21	− 6369.20; 22,679.62
1	Tx Naïve	With Cirrhosis	SOF PEG RBV 12 wk. vs BOC PEG RBV 48 wk	2	$14,343.01	2811.59; 25,874.43
1	Tx Experienced	All Patients	SOF PEG RBV 12 wk. vs PEG RBV 48 wk	2	$16,338.76	− 9294.13; 41,971.65
1	Tx Experienced	With Cirrhosis	SOF PEG RBV 12 wk. vs PEG RBV 48 wk	2	$9308.71	− 6564.02; 25,181.44
1	Tx Naïve	With Cirrhosis	SOF PEG RBV 12 wk. vs PEG RBV 48 wk	2	$14,480.59	10,944.65; 18,016.53
1	Tx Naïve	With Cirrhosis	SOF RBV 24 wk. vs No Tx	2	$45,242.13	19,014.80; 71,469.46
2	Tx Experienced	All Patients	SOF RBV 12 wk. vs No Tx	2	$16,839.10	13,661.57; 20,016.63
2	Tx Experienced	With Cirrhosis	SOF RBV 12 wk. vs No Tx	2	$20,559.84	5250.75; 35,868.93
2	Tx Naïve	With Cirrhosis	SOF RBV 12 wk. vs No Tx	2	$12,953.61	3499.67; 22,407.55
2	Tx Naïve	Without Cirrhosis	SOF RBV 12 wk. vs No Tx	2	$31,518.88	− 3263.39; 66,301.15
2	Tx Naïve	With Cirrhosis	SOF RBV 12 wk. vs PEG RBV 24 wk	2	$33,523.44	21,330.96; 45,715.92
3	Tx Experienced	All Patients	SOF PEG RBV 12 wk. vs No Tx	2	$16,543.85	9323.35; 23,764.35
3	Tx Naïve	Without Cirrhosis	SOF PEG RBV 12 wk. vs PEG RBV 24 wk	2	$162,539.68	−43,346.34; 368,425.70
3	Tx Experienced	Without Cirrhosis	SOF RBV 24 wk. vs No Tx	2	$87,973.28	− 4537.35; 180,483.91
3	Tx Naïve	Without Cirrhosis	SOF RBV 24 wk. vs No Tx	2	$52,992.71	20,221.61; 85,763.81
3	Tx Naïve	All Patients	SOF RBV 24 wk. vs PEG RBV 24 wk	2	$97,028.01	45,661.88; 148,394.14
3	Tx Naïve	With Cirrhosis	SOF RBV 24 wk. vs PEG RBV 24 wk	2	$28,155.70	15,997.15; 40,314.25
3	Tx Naïve	Without Cirrhosis	SOF RBV 24 wk. vs PEG RBV 24 wk	2	$200,289.75	94,317.94; 306,261.56
3	Tx Experienced	All Patients	SOF RBV 24 wk. vs PEG RBV 48 wk	2	$108,643.85	9097.03; 208,190.67
1	Tx Experienced	All Patients	SOF PEG RBV 12 wk. vs BOC PEG RBV 48 wk	1	$42,691.76	NA
1	Tx Experienced	Without Cirrhosis	SOF PEG RBV 12 wk. vs BOC PEG RBV 48 wk	1	$20,556.36	NA
1	Tx Experienced	All Patients	SOF PEG RBV 12 wk. vs No Tx	1	$16,636.38	NA
1	Tx Naïve	All Patients	SOF PEG RBV 12 wk. vs No Tx	1	$6183.71	NA
1	Tx Naïve	With Cirrhosis	SOF PEG RBV 12 wk. vs No Tx	1	$17,319.03	NA
1	Tx Naïve	Without Cirrhosis	SOF PEG RBV 12 wk. vs No Tx	1	$2075.11	NA
1	Tx Experienced	Without Cirrhosis	SOF PEG RBV 12 wk. vs PEG RBV 48 wk	1	$28,230.10	NA
1	Tx Naïve	Without Cirrhosis	SOF PEG RBV 12 wk. vs PEG RBV 48 wk	1	$27,395.56	NA
1	Tx Experienced	All Patients	SOF PEG RBV 12 wk. vs TEL PEG RBV 48 wk	1	$81,887.61	NA
1	Tx Experienced	With Cirrhosis	SOF PEG RBV 12 wk. vs TEL PEG RBV 48 wk	1	$30,799.23	NA
1	Tx Experienced	Without Cirrhosis	SOF PEG RBV 12 wk. vs TEL PEG RBV 48 wk	1	$22,125.61	NA
1	Tx Naïve	With Cirrhosis	SOF PEG RBV 12 wk. vs TEL PEG RBV 48 wk	1	$24,022.23	NA
1	Tx Naïve	Without Cirrhosis	SOF RBV 24 wk. vs No Tx	1	$74,046.69	NA
1	Tx Naïve	All Patients	SOF RBV 24 wk. vs PEG RBV 48 wk	1	$203,337.60	NA
2	Tx Naïve	With Cirrhosis	SOF PEG RBV 12 wk. vs PEG RBV 24 wk	1	$36,118.69	NA
2	Tx Naïve	Without Cirrhosis	SOF PEG RBV 12 wk. vs PEG RBV 24 wk	1	$242,147.86	NA
2	Tx Experienced	Without Cirrhosis	SOF RBV 12 wk. vs No Tx	1	$64,437.10	NA
2	Tx Naïve	Without Cirrhosis	SOF RBV 12 wk. vs PEG RBV 24 wk	1	$89,048.45	NA
2	Tx Experienced	With Cirrhosis	SOF RBV 12 wk. vs PEG RBV 48 wk	1	$18,783.93	NA
3	Tx Experienced	With Cirrhosis	SOF PEG RBV 12 wk. vs No Tx	1	$12,001.35	NA
3	Tx Experienced	With Cirrhosis	SOF PEG RBV 12 wk. vs PEG RBV 48 wk	1	$3389.07	NA
3	Tx Experienced	Without Cirrhosis	SOF PEG RBV 12 wk. vs PEG RBV 48 wk	1	$19,301.90	NA
3	Tx Experienced	With Cirrhosis	SOF RBV 12 wk. vs No Tx	1	$75,409.37	NA
3	Tx Experienced	Without Cirrhosis	SOF RBV 12 wk. vs No Tx	1	$186,528.47	NA
3	Tx Naïve	With Cirrhosis	SOF RBV 12 wk. vs No Tx	1	$39,799.39	NA
3	Tx Naïve	Without Cirrhosis	SOF RBV 12 wk. vs No Tx	1	$58,784.73	NA
3	Tx Naïve	With Cirrhosis	SOF RBV 12 wk. vs PEG RBV 24 wk	1	$189,241.61	NA
3	Tx Experienced	With Cirrhosis	SOF RBV 24 wk. vs PEG RBV 48 wk	1	$70,111.60	NA
3	Tx Experienced	Without Cirrhosis	SOF RBV 24 wk. vs PEG RBV 48 wk	1	$58,828.37	NA

Key: *BOC* = boceprevir; *NA* = not applicable, *PEG* = pegylated interferon, *RBV* = ribavirin, *SOF* = sofosbuvir; *Tx* = treatment, *vs* = versus, *wk*. = week, *95% CI*, *95%* confidence interval “lower bound; upper bound”

Based on data reported in included intervention models: Cure 2015a [28], Cure 2015b [29], Linas, 2015 [37], Liu 2014 [39], Najafzadeh 2015 [41], San Miguel 2015 [47], Saab 2014 [46]

As we took into consideration the different comparisons (intervention and comparator) for the calculation of ICERs, the synthetized values are all based in unique comparisons. All comparisons are shown in the column labelled “Treatment” in Table 6.

The outcome of all studies was cost per quality-adjusted life-years (QALY), a measure that represents the cost incurred for gaining one year of life adjusted for the quality of life. Due to many comparisons and a small sample, a great variability was found in the ICERs (large coefficient of variation). Half (55) of the combinations resulted in mean ICERs above $30,000. The other half (with ICERs below $30,000) was tested 62 times overall. Approximately 27% of the ICER estimates suggested that DAAs were not cost-effective considering a threshold of $50,000 (see Table 6).

#### Surveillance

A meta-analysis of the results from these models was not possible due to the different surveillance strategies compared a result of the small number of included studies. As a result, we present a narrative summary of the results from the models identified.

In the model presented by Liu et al. (2011), results indicated that early treatment of CHC can be the cost-effective strategy compared to the implementation of testing approaches [57]. However, for clinical settings where testing is required prior to treatment, FibroTest® only was more effective and also less costly than liver biopsy [57].

The model by Canavan et al. (2013) demonstrated that a strategy of annual definitive Fibroscan® TE diagnosed 20% more cirrhosis cases than the current strategy, with 549 extra patients per 10,000 accessing screening over a lifetime and, consequently, 76 additional HCC cases diagnosed [56].

In the third model identified (Crossan et al., 2015), the authors concluded that when applying the standard UK cost-effectiveness threshold range, the cost-effective strategy was a “treat all” approach resulting in an ICER of £9204 [54]. In the same direction, a research published in 2011, with the payer perspective in USA, proposed a “shift towards strategies that initiate immediate treatment without fibrosis screening” [57].

In summary, the findings of two [54, 57] of the three models evaluating surveillance strategies suggest that treating all CHC patients regardless of the staging of liver disease, could be cost-effective. These analyses were conducted according to the perspectives of USA third party payer (direct healthcare costs only), and the UK National Health Service.

## Discussion

This systematic review was able to identify and analyse studies and model structures and parameters used to estimate the cost-effectiveness of surveillance or treatment of people living with CHC. The review demonstrated that of the eight intervention studies that we evaluated in detail, very similar model structures were used to investigate the cost-effectiveness of DAA treatments for CHC.

### Models

The majority of included models adopted a Markov approach [28, 29, 39, 44, 46, 47] with METAVIR-based classification used as health states [39, 41, 46]. The presence of cirrhosis (with/without) was another important factor for model structures, with some models including this characteristic as a separate health state [28, 29, 37, 44, 47]. A lifetime time horizon was used in the majority of cases [28, 29, 37, 39, 41, 44, 47], but cycle length ranged from 30 days [37] to 12 months [44, 46]. All the differences found in model structures and cycle length can impose limitation to comparability of the studies and to stakeholder’s decision. The variation found in cycle length might have clinical implication for the results, as the HCV treatment time is being shortened with the use of new drugs. Populations with different characteristics were analysed (e.g.: HCV genotype; prior treatment status (naïve or experienced); cirrhotic or non-cirrhotic; HIV coinfection). Moreover, a number of different treatment comparisons (several drugs and treatment durations) were used for the included studies.

### Quality appraisal

The included studies were quality appraised using two checklists (CHEERS and Philips [14, 15]). This assessment identified a number of issues, largely a result of reporting omissions. Accordingly, the major finding of the results of our study is that modelling process should be better described, especially considering model validation and calibration. The implications of these unclear descriptions are that the results can be biased and the decisions made on their basis cannot achieve what was expected. Consequently, real life outcomes might be much different from modelling results, producing unexpected additional budget impact for the health system. Studies should better report their modelling process.

We have not enough data to state if the results obtained by microsimulation models or cohort Markov structure were better. Although discrete event simulation can be considered more powerful in terms of capacity of reflecting real-world changes, the memory-less assumption of the Markov model is not a critical issue for CHC.

A good reason to explain the variation in the results is the use of different data from different settings and perspectives, this issue is as critical as the model structures in term of producing variability. We argue that model structures were relative common among the different studies, and it was not possible to identify any study with insufficient modelling structure.

### Data synthesis

The ICERs of treatment were quantitatively synthesized. The cost-effectiveness results for treatment and surveillance indicated important differences. This heterogeneity needs to be contextualized in relation to the different populations, interventions, populations, settings and perspectives of the studies. However, we could only undertake a quantitative synthesis of the models evaluating treatments. The conducted synthesis was limited too by the number of studies that could be combined.

CEAs of HCV treatments should be discussed in relation to the considerable high variability in their ICERs estimates. This analysis suggests that in most circumstances DAAs were cost-effective (when using an ICER threshold of $50,000 per quality-adjusted life-year [QALY]). Considering that new treatments with DAAs have demonstrated high effectiveness [9, 58], the cost dimension is the main challenge for implementation worldwide. Although the cost of the new CHC drugs shows global variation some have suggested they are unaffordable [59]. However, in some countries negotiation with pharmaceutical companies has been successful in providing discounts [60]. This strategy could therefore be adopted in other settings with universal health systems, and has the potential to not only improve cost-effectiveness but increase patients’ access to the highly effective DAAs.

### Previous systematic reviews

A previously published systematic review of CEAs that evaluated DAAs found that the modelling structures were similar [12]. In that review, the quality of the included studies was reported as being acceptable by the authors that used CHEERS (reporting quality) checklist only [12]. In our systematic review, we included a second checklist and synthetized ICERs when possible. However, these synthesis results are limited by the literature search update. Regarding surveillance of CHC, a recent systematic review compared TE with liver biopsy and found it cost-effective especially for patients with a higher degree of liver fibrosis. In that review a high variability in methodological quality was found, using the Drummond 10-item checklist [10].

### Surveillance studies

Focusing on the included surveillance studies, a treat-all strategy was suggested as cost-effective by two studies [54, 57]. However, those findings can be limited to the local settings, thresholds, and also the perspectives used of USA payer and UK NHS. These conclusions may not apply to lower and middle-income countries. Surveillance and treatment prioritization for the subgroup of CHC patients with higher risk of liver disease progression can be an option. Moreover, the presence of different surveillance strategies in the included studies complicates the analysis of this systematic review. Thus, clinicians and policy makers might have similar problems to achieve the most appropriate treatment decision due to the number of alternatives to be considered.

### Clinical implications

Treatment of chronic hepatitis C was revolutionized by high efficacy of direct-acting antiviral drugs (DAAs). However, the decrease of the burden of liver disease in CHC patients by DAA treatment has been associated with high costs to health authorities worldwide [61]. The analysis of studies that evaluated cost-effectiveness of HCV eradication by DAAs is essential for elaboration of public health strategies to promote large primary care access to DAAs regimens, especially in low to middle-income countries with high CHC prevalence.

### Limitations

This paper has several limitations. Our findings, especially those related to clinical implications and ICERs synthesis, just represent the circumstances present at the moment of the last search update (May 2015); and, study selection and data extraction can impose risk of bias, even after training of the review team. Considering the limitations of the present review, the variability of the studies included is certainly a factor that should be addressed.

## Conclusions

CEAs of CHC treatments presented variability in their cost-effectiveness estimates. Our analysis suggests that there were still some circumstances where DAAs were not cost-effective. Thus surveillance, as opposed to a treat-all strategy may still need to be considered as an option for deploying DAAs, particularly where acquisition cost is at the limit of affordability for a health service. We identified existing models, which could be used to compare surveillance and treat-all strategies. Future studies should compare the cost-effectiveness of the surveillance of liver disease with a treat-all strategy for CHC patients considering different settings and perspectives.

## Additional files

Additional file 1: Search Strategies. (DOCX 16 kb)

Additional file 2: List of excluded studies, Studies by each reason for exclusion. (DOCX 43 kb)

## Abbreviations

BOC: Boceprevir
CEA(s): Cost-effectiveness analysis(ses)
CHC: Chronic hepatitis C
CI: Confidence interval
DAA(s): Direct acting anti-viral agent(s)
HCV: Hepatitis C virus
ICER(s): Incremental cost-effectiveness ratio(s)
NHS: National Health Service
PEG: Pegylated interferon
PPP: Purchasing power parity
QALY: Quality-adjusted life-year
RBV: Ribavirin
SOF: Sofosbuvir
TE: Transient elastography
Tx: Treatment
UCICT: Unique combination of intervention versus comparator with time in weeks of treatment duration
UK: United Kingdom
USA: United States of America
vs: Versus
wk.: Week

## References

[1] Shirachi R, Shiraishi H, Tateda A, Kikuchi K, Ishida N (1978). Hepatitis "C" antigen in non-a, non-B post-transfusion hepatitis. Lancet.

[2] Houghton M, Weiner A, Han J, Kuo G, Choo QL (1991). Molecular biology of the hepatitis C viruses: implications for diagnosis, development and control of viral disease. Hepatology.

[3] Clarke A, Kulasegaram R (2006). Hepatitis C transmission -- where are we now?. Int J STD AIDS.

[4] Matthews GV, Pham ST, Hellard M, Grebely J, Zhang L, Oon A, Marks P, van Beek I, Rawlinson W, Kaldor JM (2011). Patterns and characteristics of hepatitis C transmission clusters among HIV-positive and HIV-negative individuals in the Australian trial in acute hepatitis C. Clin Infect Dis.

[5] Westbrook RH, Dusheiko G (2014). Natural history of hepatitis C. J Hepatol.

[6] Wedemeyer H, Dore GJ, Ward JW (2015). Estimates on HCV disease burden worldwide - filling the gaps. J Viral Hepat.

[7] Innes H, Goldberg D, Dillon J, Hutchinson SJ (2015). Strategies for the treatment of hepatitis C in an era of interferon-free therapies: what public health outcomes do we value most?. Gut.

[8] Ward JW, Mermin JH (2015). Simple, effective, but out of reach? Public health implications of HCV drugs. N Engl J Med.

[9] Sulkowski MS, Vargas HE, Di Bisceglie AM, Kuo A, Reddy KR, Lim JK, Morelli G, Darling JM, Feld JJ, Brown RS (2016). Effectiveness of Simeprevir plus Sofosbuvir, with or without ribavirin, in real-world patients with HCV genotype 1 infection. Gastroenterology.

[10] van Katwyk S, Coyle D, Cooper C, Pussegoda K, Cameron C, Skidmore B, Brener S, Moher D, Thavorn K. Transient elastography for the diagnosis of liver fibrosis: a systematic review of economic evaluations. Liver Int. 2017;37(6):851–61 10.1111/liv.13260 27699993

[11] Gentile I, Maraolo AE, Niola M, Graziano V, Borgia G, Paternoster M (2016). Limiting the access to direct-acting antivirals against HCV: an ethical dilemma. Expert Rev Gastroenterol Hepatol.

[12] Chhatwal J, He T, Lopez-Olivo MA (2016). Systematic review of modelling approaches for the cost effectiveness of hepatitis C treatment with direct-acting antivirals. Pharmacoeconomics.

[13] Centre for Reviews and Dissemination (2009). Systematic Reviews - CRD’s guidance for undertaking reviews in health care. Centre for Reviews and Dissemination.

[14] Philips Z, Bojke L, Sculpher M, Claxton K, Golder S (2006). Good practice guidelines for decision-analytic modelling in health technology assessment: a review and consolidation of quality assessment. Pharmacoeconomics.

[15] Husereau D, Drummond M, Petrou S, Carswell C, Moher D, Greenberg D, Augustovski F, Briggs AH, Mauskopf J, Loder E (2013). Consolidated health economic evaluation reporting standards (CHEERS) statement. Value in health J International Society for Pharmacoeconomics Outcomes Res.

[16] R Core Team: R: A language and environment for statistical computing. In*.* Vienna, Austria: R Foundation for Statistical Computing; 2017: URL https://www.r-project.org/. 24 May 2018.

[17] Athanasakis K, Ferrante SA, Kyriopoulos II, Petrakis I, Hill M, Retsa MP, Kyriopoulos J (2015). Boceprevir for chronic genotype 1 hepatitis C virus in the current health care setting in Greece: a cost-effectiveness analysis. Clin Ther.

[18] Blázquez-Pérez A, San Miguel R, Mar J (2013). Cost-effectiveness analysis of triple therapy with protease inhibitors in treatment-naive hepatitis C patients. Pharmacoeconomics.

[19] Brogan AJ, Talbird SE, Thompson JR, Miller JD, Rubin J, Deniz B (2014). Cost-effectiveness of telaprevir combination therapy for chronic hepatitis C. PLoS One.

[20] Cammà C, Petta S, Cabibbo G, Ruggeri M, Enea M, Bruno R, Capursi V, Gasbarrini A, Alberti A, Craxì A (2013). Cost-effectiveness of boceprevir or telaprevir for previously treated patients with genotype 1 chronic hepatitis C. J Hepatol.

[21] Cammà C, Petta S, Enea M, Bruno R, Bronte F, Capursi V, Cicchetti A, Colombo GL, Di Marco V, Gasbarrini A (2012). Cost-effectiveness of boceprevir or telaprevir for untreated patients with genotype 1 chronic hepatitis C. Hepatology.

[22] Chan K, Lai MN, Groessl EJ, Hanchate AD, Wong JB, Clark JA, Asch SM, Gifford AL, Ho SB (2013). Cost effectiveness of direct-acting antiviral therapy for treatment-naive patients with chronic HCV genotype 1 infection in the veterans health administration. Clin Gastroenterol Hepatol.

[23] Chhatwal J, Ferrante SA, Brass C, El Khoury AC, Burroughs MBB, Esteban-Mur R, Elbasha EH. Cost-effectiveness of Boceprevir in patients previously treated for chronic hepatitis C genotype 1 infection in the United States. Value Health. 2013;16(6) 10.1016/j.jval.2013.1007.1006. 10.1016/j.jval.2013.07.006 PMC3820000 24041347

[24] Chhatwal J, Kanwal F, Roberts MS, Dunn MA (2015). Cost-effectiveness and budget impact of hepatitis C virus treatment with Sofosbuvir and Ledipasvir in the United States. Ann Intern Med.

[25] Cortesi PA, Mantovani LG, Ciaccio A, Rota M, Mazzarelli C, Cesana G, Strazzabosco M, Belli LS (2015). Cost-effectiveness of new direct-acting antivirals to prevent post–liver transplant recurrent hepatitis. Am J Transplant Off J Am Soc Transplant Am Soc Transplant Surg.

[26] Cure S, Bianic F, Gavart S, Curtis S, Lee S, Dusheiko G (2014). Cost-effectiveness of telaprevir in combination with pegylated interferon alpha and ribavarin in previously untreated chronic hepatitis C genotype 1 patients. J Med Econ.

[27] Cure S, Bianic F, Gavart S, Curtis S, Lee S, Dusheiko G (2014). Cost-effectiveness of telaprevir in combination with pegylated interferon alpha and ribavarin in treatment-experienced chronic hepatitis C genotype 1 patients. J Med Econ.

[28] Cure S, Guerra I, Dusheiko G (2015). Cost-effectiveness of sofosbuvir for the treatment of chronic hepatitis C-infected patients. J Viral Hepat.

[29] Cure S, Guerra I, Camma C, Craxi A, Carosi G (2015). Cost-effectiveness of sofosbuvir plus ribavirin with or without pegylated interferon for the treatment of chronic hepatitis C in Italy. J Med Econ.

[30] Dan YY, Ferrante SA, Elbasha EH, Hsu TY (2015). Cost-effectiveness of boceprevir co-administration versus pegylated interferon-alpha2b and ribavirin only for patients with hepatitis C genotype 1 in Singapore. Antivir Ther.

[31] Elbasha EH, Chhatwal J, Ferrante SA, El Khoury AC, Laires PA (2013). Cost-effectiveness analysis of Boceprevir for the treatment of chronic hepatitis C virus genotype 1 infection in Portugal. Applied Health Economics and Health Policy.

[32] Ferrante SA, Chhatwal J, Brass CA, El Khoury AC, Poordad F, Bronowicki J-P, Elbasha EH (2013). Boceprevir for previously untreated patients with chronic hepatitis C genotype 1 infection: a US-based cost-effectiveness modeling study. BMC Infect Dis.

[33] Gimeno-Ballester V, Mar J, San Miguel R (2016). Cost–effectiveness analysis of simeprevir with daclatasvir for non-cirrhotic genotype-1b-naïve patients plus chronic hepatitis C. Expert Rev Pharmacoecon Outcomes Res.

[34] Hagan LM, Sulkowski MS, Schinazi RF (2014). Cost analysis of sofosbuvir/ribavirin versus sofosbuvir/simeprevir for genotype 1 hepatitis C virus in interferon-ineligible/intolerant individuals. Hepatology.

[35] Leleu H, Blachier M, Rosa I (2015). Cost-effectiveness of sofosbuvir in the treatment of patients with hepatitis C. J Viral Hepat.

[36] Linas BP, Barter DM, Leff JA, Di Lorenzo M, Schackman BR, Horsburgh RC, Assoumou SA, Salomon JA, Weinstein MC, Kim AY (2014). The cost-effectiveness of improved HCV therapies in HIV/HCV co-infected individuals. AIDS (London, England).

[37] Linas BP, Barter DM, Morgan JR, Pho MT, Leff JA, Schackman BR, Horsburgh CR, Assoumou SA, Salomon JA, Weinstein MC (2015). The cost-effectiveness of sofosbuvir-based regimens for treatment of hepatitis C virus genotype 2 or 3 infection. Ann Intern Med.

[38] Liu S, Cipriano LE, Holodniy M, Owens DK, Goldhaber-Fiebert JD (2012). New protease inhibitors for the treatment of chronic hepatitis C: a cost-effectiveness analysis. Ann Intern Med.

[39] Liu S, Watcha D, Holodniy M, Goldhaber-Fiebert JD (2014). Sofosbuvir-based treatment regimens for chronic, genotype 1 hepatitis C virus infection in U.S. incarcerated populations: a cost-effectiveness analysis. Ann Intern Med.

[40] McEwan P, Ward T, Bennett H, Kalsekar A, Webster S, Brenner M, Yuan Y (2015). Estimating the clinical and economic benefit associated with incremental improvements in sustained Virologic response in chronic hepatitis C. PLoS One.

[41] Najafzadeh M, Andersson K, Shrank WH, Krumme AA, Matlin OS, Brennan T, Avorn J, Choudhry NK (2015). Cost-effectiveness of novel regimens for the treatment of hepatitis C virus. Ann Intern Med.

[42] Petta S, Cabibbo G, Enea M, Macaluso FS, Plaia A, Bruno R, Gasbarrini A, Bruno S, Craxì A, Cammà C (2014). Personalized cost-effectiveness of boceprevir-based triple therapy for untreated patients with genotype 1 chronic hepatitis C. Dig Liver Dis.

[43] Petta S, Cabibbo G, Enea M, Macaluso FS, Plaia A, Bruno R, Gasbarrini A, Craxì A, Cammà C (2014). Cost-effectiveness of sofosbuvir-based triple therapy for untreated patients with genotype 1 chronic hepatitis C. Hepatology.

[44] Pfeil AM, Reich O, Guerra IM, Cure S, Negro F, Mullhaupt B, Lavanchy D, Schwenkglenks M (2015). Cost-effectiveness analysis of sofosbuvir compared to current standard treatment in Swiss patients with chronic hepatitis C. PLoS One.

[45] Rein DB, Wittenborn JS, Smith BD, Liffmann DK, Ward JW (2015). The cost-effectiveness, health benefits, and financial costs of new antiviral treatments for hepatitis C virus. Clin Infect Dis.

[46] Saab S, Gordon SC, Park H, Sulkowski M, Ahmed A, Younossi Z (2014). Cost-effectiveness analysis of sofosbuvir plus peginterferon/ribavirin in the treatment of chronic hepatitis C virus genotype 1 infection. Aliment Pharmacol Ther.

[47] San Miguel R, Gimeno-Ballester V, Blazquez A, Mar J: Cost-effectiveness analysis of sofosbuvir-based regimens for chronic hepatitis C (Provisional abstract). In: Gut 2014: epub. 10.1136/gutjnl-2014-307772 25311032

[48] Tice A, Ollendorf DA, Pearson SD (2014). The comparative clinical effectiveness and value of simeprevir and sofosbuvir in the treatment of chronic hepatitis C infection, a technology assessment (final report). USA: Institute for Clinical and Economic Review (California technology assessment Forum).

[49] Vellopoulou A, van Agthoven M, van der Kolk A, de Knegt RJ, Berdeaux G, Cure S, Bianic F, Lamotte M (2014). Cost utility of Telaprevir–PR (Peginterferon–ribavirin) versus Boceprevir–PR and versus PR alone in chronic hepatitis C in the Netherlands. Applied Health Economics and Health Policy.

[50] Warren E, Wright A, Jones B (2014). Cost-effectiveness of Telaprevir in patients with genotype 1 hepatitis C in Australia. Value Health.

[51] Westerhout K, Treur M, Mehnert A, Pascoe K, Ladha I, Belsey J (2015). A cost utility analysis of simeprevir used with peginterferon + ribavirin in the management of genotype 1 hepatitis C virus infection, from the perspective of the UK national health service. J Med Econ.

[52] Younossi ZM, Park H, Saab S, Ahmed A, Dieterich D, Gordon SC (2015). Cost-effectiveness of all-oral ledipasvir/sofosbuvir regimens in patients with chronic hepatitis C virus genotype 1 infection. Aliment Pharmacol Ther.

[53] Zhang S, Bastian ND, Griffin PM (2015). Cost-effectiveness of sofosbuvir-based treatments for chronic hepatitis C in the US. BMC Gastroenterol.

[54] Crossan C, Tsochatzis EA, Longworth L, Gurusamy K, Davidson B, Rodriguez-Peralvarez M, Mantzoukis K, O'Brien J, Thalassinos E, Papastergiou V (2015). Cost-effectiveness of non-invasive methods for assessment and monitoring of liver fibrosis and cirrhosis in patients with chronic liver disease: systematic review and economic evaluation. Health Technol Assess.

[55] Tsochatzis EA, Crossan C, Longworth L, Gurusamy K, Rodriguez-Peralvarez M, Mantzoukis K, O'Brien J, Thalassinos E, Papastergiou V, Noel-Storr A (2014). Cost-effectiveness of noninvasive liver fibrosis tests for treatment decisions in patients with chronic hepatitis C. Hepatology.

[56] Canavan C, Eisenburg J, Meng L, Corey K, Hur C (2013). Ultrasound elastography for fibrosis surveillance is cost effective in patients with chronic hepatitis C virus in the UK. Dig Dis Sci.

[57] Liu S, Schwarzinger M, Carrat F, Goldhaber-Fiebert JD (2011). Cost effectiveness of fibrosis assessment prior to treatment for chronic hepatitis C patients. PLoS One.

[58] Pol S, Bourliere M, Lucier S, Hezode C, Dorival C, Larrey D, Bronowicki JP, Ledinghen VD, Zoulim F, Tran A (2017). Safety and efficacy of daclatasvir-sofosbuvir in HCV genotype 1-mono-infected patients. J Hepatol.

[59] Iyengar S, Tay-Teo K, Vogler S, Beyer P, Wiktor S, de Joncheere K, Hill S (2016). Prices, costs, and affordability of new medicines for hepatitis C in 30 countries: an economic analysis. PLoS Med.

[60] Mesquita F, Santos ME, Benzaken A, Corrêa RG, Cattapan E, Sereno LS, Naveira MCM (2016). The Brazilian comprehensive response to hepatitis C: from strategic thinking to access to interferon-free therapy. BMC Public Health.

[61] Ward T, Gordon J, Bennett H, Webster S, Sugrue D, Jones B, Brenner M, McEwan P (2016). Tackling the burden of the hepatitis C virus in the UK: characterizing and assessing the clinical and economic consequences. Public Health.

