# Supplementary material for: Cost-effectiveness of diagnostic and therapeutic interventions for chronic hepatitis C: a systematic review of model-based analyses
Source: BMC Med Res Methodol. 2018 Jun 13;18:53. doi: 10.1186/s12874-018-0515-9 (PMC5998601; doi:10.1186/s12874-018-0515-9)
Supplement: Supplementary file 1 — Search Strategies. (DOCX 16 kb) [file 12874_2018_515_MOESM1_ESM.docx]

**Additional file 1: Search Strategies.**

| **Database** | **Hits** |
| --- | --- |
| MEDLINE and MEDLINE in Process | 1231 |
| EMBASE | 2071 |
| NHS EEDs (The Cochrane Library) | 403 |
| HTA Library (The Cochrane Library) | 28 |
| Lilacs | 25 |
| Total | 3758 |
| -duplicates | 1355 |
| unique studies to screen | 2403 |

**Database(s):** Ovid MEDLINE(R) In-Process & Other Non-Indexed Citations and Ovid MEDLINE(R)

**Host:** OVID

**Data Parameters:** 1946 to Present

**Date Searched:** 12/05/2015

**Information Specialist:** CC

**Searcher:** RC(Press Checked: HP)

| **#** | **Searches** | **Results** |
| --- | --- | --- |
| 1 | exp Hepatitis C/ | 50119 |
| 2 | (hepatitis c or Hepatitis C or hepacivir$ or (chronic adj3 hepatitis)).ti,ab,kw,ot. | 81035 |
| 3 | 1 or 2 | 90939 |
| 4 | ((economic adj3 evaluat$) or (cost$ adj3 (utility or decision or benefit or model or effect$ or minimisation or minimization)) or (CBA or CEA or CUA) or DAM or (decision adj3 (model$ or analytic or tree)) or (model based or model-based) or Pharmacoeconomics).ti,ab,kw,ot. | 171572 |
| 5 | exp *decision support techniques/ | 18667 |
| 6 | 4 or 5 | 188299 |
| 7 | 3 and 6 | 1231 |

**Host:** OVID

**Data Parameters:** 1974 to 2015 May 11

**Date Searched:** 12/05/2015

**Information Specialist:** CC

**Searcher:** RC (Press Checked: HP)

**Database(s):** Embase 1974 to 2015 May 11

| **#** | **Searches** | **Results** |
| --- | --- | --- |
| 1 | exp hepatitis C/ | 79621 |
| 2 | (hepatitis c or Hepatitis C or hepacivir$ or (chronic adj3 hepatitis)).ti,ab,kw,ot. | 112761 |
| 3 | 1 or 2 | 132467 |
| 4 | ((economic adj3 evaluat$) or (cost$ adj3 (utility or decision or benefit or model or effect$ or minimisation or minimization)) or (CBA or CEA or CUA) or DAM or (decision adj3 (model$ or analytic or tree)) or (model based or model-based) or Pharmacoeconomics).ti,ab,kw,ot. | 225980 |
| 5 | *decision support system/ | 7652 |
| 6 | 4 or 5 | 232730 |
| 7 | 3 and 6 | 2071 |

**NHS Economic Evaluation Database:** Issue 1 of 4, January 2015

**Search Name:**

**Date Run:** 24/03/15 16:59:42.455

**Description:** Technology Assessments (n = 28) / Economic Evaluations (n = 403)

**Information Specialist:** CC

**Searcher:**  RC (Press Checked: HP)

| **#** | **Searches** | **Results** |
| --- | --- | --- |
| #1 | MeSH descriptor: [Hepatitis C] explode all trees | 2338 |
| #2 | (hepatitis c or Hepatitis C or hepacivir* or (chronic near/2 hepatitis)) | 9401 |
| #3 | #1 or #2 | 9401 |
| #4 | ((economic near/2 evaluat*) or (cost* near/2 (utility or decision or benefit or model or effect* or minimisation or minimization)) or (CBA or CEA or CUA) or DAM or (decision near/2 (model* or analytic or tree)) or (model based or model-based) or Pharmacoeconomics) | 52320 |
| #5 | MeSH descriptor: [Decision Support Techniques] this term only | 1611 |
| #6 | #4 or #5 | 52672 |
| #7 | #3 and #6 | 1206 |

**Database(s):** Lilacs

**Data Parameters:** 1998 to 2014

**Date Searched:** 12/05/2015

**Information Specialist:** CC

**Searcher:** RC (Press Checked: HP)

**Search Strategy:** Lilacs (n = 25)

tw:("Hepatitis C" AND (((economic adj3 evaluat*) OR (cost* adj3 (utility OR decision OR benefit OR model OR effect* OR minimisation OR minimization)) OR (cba OR cea OR cua) OR dam OR (decision adj3 (model* OR analytic OR tree)) OR (model based OR model-based) OR pharmacoeconomics))) AND ( db:("LILACS"))
