# Supplementary material for: Cost-effectiveness of diagnostic and therapeutic interventions for chronic hepatitis C: a systematic review of model-based analyses
Source: BMC Med Res Methodol. 2018 Jun 13;18:53. doi: 10.1186/s12874-018-0515-9 (PMC5998601; doi:10.1186/s12874-018-0515-9)
Supplement: Supplementary file 2 — List of excluded studies, Studies by each reason for exclusion. (DOCX 43 kb) [file 12874_2018_515_MOESM2_ESM.docx]

**Additional file 2: List of excluded studies**

**Studies by each reason for exclusion.**

**Reason for exclusion, population (n=3)**

BARTOLOMEO, N., TREROTOLI, P. & SERIO, G. 2011. Progression of liver cirrhosis to HCC: an application of hidden Markov model. BMC Med Res Methodol, 11, 38.

PEREZ, R. U., CASTILLO MUNOZ, M. A., NAVARRO CABALLERO, J. A. & MARQUEZ PELAEZ, S. 2012. Inhibidores de la proteasa (boceprevir y telaprevir) en el tratamiento de pacientes monoinfectados por el VHC: eficacia, seguridad y eficiencia comparada [Protease inhibitors (Boceprevir and telaprevir) in the treatment of chronic HCV infection: relative efficacy, safety and efficiency]. York (UK): CRD York HTA Database.

THOMPSON COON, J., ROGERS, G., HEWSON, P., WRIGHT, D., ANDERSON, R., JACKSON, S., RYDER, S., CRAMP, M. & STEIN, K. 2008. Surveillance of cirrhosis for hepatocellular carcinoma: a cost-utility analysis. Br J Cancer, 98, 1166-75.

**Reason for exclusion, intervention (n=127)**

ANNEMANS, L., WARIE, H., NECHELPUT, M. & PERAUX, B. 2004. A health economic model to assess the long term effects and cost-effectiveness of PEG IFN alpha-2a in hepatitis C virus infected patients. Acta Gastroenterol Belg, 67, 1-8.

BARROS, F. M., CHEINQUER, H., TSUCHIYA, C. T. & SANTOS, E. A. 2013. Cost-effectiveness analysis of treatment with peginterferon-alfa-2a versus peginterferon-alfa-2b for patients with chronic hepatitis C under the public payer perspective in Brazil. Cost Eff Resour Alloc, 11, 25.

BENNETT, W. G., INOUE, Y., BECK, J. R., WONG, J. B., PAUKER, S. G. & DAVIS, G. L. 1997. Estimates of the cost-effectiveness of a single course of interferon-alpha 2b in patients with histologically mild chronic hepatitis C. Ann Intern Med, 127, 855-65.

BERNFORT, L., SENNFALT, K. & REICHARD, O. 2006. Cost-effectiveness of peginterferon alfa-2b in combination with ribavirin as initial treatment for chronic hepatitis C in Sweden. Scand J Infect Dis, 38, 497-505.

BLATT, C. R., STORB, B., MUHLBERGER, N., FARIAS, M. R. & SIEBERT, U. 2014. Chronic hepatitis C treatment for genotype 2 or 3 in Brazil: cost effectiveness analysis of peginterferon plus ribavarin as first choice treatment. Brazilian Journal of Pharmaceutical Sciences, 50, 345-352.

BOCK, J. A., FAIRLEY, K. J., SMITH, R. E., MAENG, D. D., PITCAVAGE, J. M., INVERSO, N. A. & WILLIAMS, M. S. 2014. Cost-effectiveness of IL28Beta genotype-guided protease inhibitor triple therapy versus standard of care treatment in patients with hepatitis C genotypes 2 or 3 infection. Public Health Genomics, 17, 306-19.

BUTI, M., CASADO, M. A. & ESTEBAN, R. 2007. Evaluating the cost of sustained virologic response in naive chronic hepatitis C patients treated a la carte. Aliment Pharmacol Ther, 26, 705-16.

BUTI, M., CASADO, M. A., FOSBROOK, L. & ESTEBAN, R. 2002. Which is the most cost effective combination therapy strategy using interferon alpha-2b plus ribavarin for naive patients with chronic hepatitis C? Clinical Drug Investigation, 22, 31-39.

BUTI, M., CASADO, M. A., FOSBROOK, L. & ESTEBAN, R. 2005. Financial impact of two different ways of evaluating early virological response to peginterferon-alpha-2b plus ribavirin therapy in treatment-naive patients with chronic hepatitis C virus genotype 1. Pharmacoeconomics, 23, 1043-55.

BUTI, M., CASADO, M. A., FOSBROOK, L., WONG, J. B. & ESTEBAN, R. 2000. Cost-effectiveness of combination therapy for naive patients with chronic hepatitis C. J Hepatol, 33, 651-8.

BUTI, M., MEDINA, M., CASADO, M. A., WONG, J. B., FOSBROOK, L. & ESTEBAN, R. 2003. A cost-effectiveness analysis of peginterferon alfa-2b plus ribavirin for the treatment of naive patients with chronic hepatitis C. Aliment Pharmacol Ther, 17, 687-94.

DAVIS, G. L., BECK, J. R., FARRELL, G. & POYNARD, T. 1998. Prolonged treatment with interferon in patients with histologically mild chronic hepatitis C: a decision analysis. J Viral Hepat, 5, 313-21.

DEUFFIC-BURBAN, S., BABANY, G., LONJON-DOMANEC, I., DELTENRE, P., CANVA-DELCAMBRE, V., DHARANCY, S., LOUVET, A., ROUDOT-THORAVAL, F. & MATHURIN, P. 2009. Impact of pegylated interferon and ribavirin on morbidity and mortality in patients with chronic hepatitis C and normal aminotransferases in France. Hepatology, 50, 1351-9.

DEUFFIC-BURBAN, S., DELTENRE, P., BUTI, M., STROFFOLINI, T., PARKES, J., MUHLBERGER, N., SIEBERT, U., MORENO, C., HATZAKIS, A., ROSENBERG, W., ZEUZEM, S. & MATHURIN, P. 2012. Predicted effects of treatment for HCV infection vary among European countries. Gastroenterology, 143, 974-85 e14.

DOLDER, N. M., WILHARDT, M. S. & MORREALE, A. P. 2002. Justifying a multidisciplinary high-intensity hepatitis C clinic by using decision analysis. Am J Health Syst Pharm, 59, 867-71.

DUSHEIKO, G. M. & ROBERTS, J. A. 1995. Treatment of chronic type B and C hepatitis with interferon alfa: an economic appraisal. Hepatology, 22, 1863-73.

EL HOUBY, E. M. 2014. A framework for prediction of response to HCV therapy using different data mining techniques. Adv Bioinformatics, 2014, 181056.

EL SAADANY, S., COYLE, D., GIULIVI, A. & AFZAL, M. 2005. Economic burden of hepatitis C in Canada and the potential impact of prevention. Results from a disease model. Eur J Health Econ, 6, 159-65.

FONSECA, M. C., ARAUJO, G. T. & ARAUJO, D. V. 2009. Cost effectiveness of peginterferon alfa-2B combined with ribavirin for the treatment of chronic hepatitis C in Brazil. Braz J Infect Dis, 13, 191-9.

GARCIA DE ANCOS, J. L., ROBERTS, J. A. & DUSHEIKO, G. M. 1990. An economic evaluation of the costs of alpha-interferon treatment of chronic active hepatitis due to hepatitis B or C virus. J Hepatol, 11 Suppl 1, S11-8.

GARCIA-CONTRERAS, F., NEVAREZ-SIDA, A., CONSTANTINO-CASAS, P., ABUD-BASTIDA, F. & GARDUNO-ESPINOSA, J. 2006. Cost-effectiveness of chronic hepatitis C treatment with thymosin alpha-1. Arch Med Res, 37, 663-73.

GELLAD, Z. F., MUIR, A. J., MCHUTCHISON, J. G., SIEVERT, W., SHARARA, A. I., BROWN, K. A., FLISIAK, R., JACOBSON, I. M., KERSHENOBICH, D., MANNS, M. P., SCHULMAN, K. A. & REED, S. D. 2012. Cost-effectiveness of truncated therapy for hepatitis C based on rapid virologic response. Value Health, 15, 876-86.

GERKENS, S., NECHELPUT, M., ANNEMANS, L., PERAUX, B., BEGUIN, C. & HORSMANS, Y. 2007. A health economic model to assess the cost-effectiveness of pegylated interferon alpha-2a and ribavirin in patients with moderate chronic hepatitis C and persistently normal alanine aminotransferase levels. Acta Gastroenterol Belg, 70, 177-87.

GERKENS, S., NECHELPUT, M., ANNEMANS, L., PERAUX, B., MOUCHART, M., BEGUIN, C. & HORSMANS, Y. 2007. A health economic model to assess the cost-effectiveness of PEG IFN alpha-2a and ribavirin in patients with mild chronic hepatitis C. J Viral Hepat, 14, 523-36.

GHEORGHE, L. & BACULEA, S. 2010. Cost-effectiveness of peginterferon alpha-2a and peginterferon alpha-2b combination regimens in genotype-1 naive patients with chronic hepatitis C. Hepatogastroenterology, 57, 939-44.

GORDON, S. C., POCKROS, P. J., TERRAULT, N. A., HOOP, R. S., BUIKEMA, A., NERENZ, D. & HAMZEH, F. M. 2012. Impact of disease severity on healthcare costs in patients with chronic hepatitis C (CHC) virus infection. Hepatology, 56, 1651-60.

GRIEVE, R. & ROBERTS, J. 2002. Economic evaluation for hepatitis C. Acta Gastroenterol Belg, 65, 104-9.

GRIEVE, R., ROBERTS, J., WRIGHT, M., SWEETING, M., DE ANGELIS, D., ROSENBERG, W., BASSENDINE, M., MAIN, J. & THOMAS, H. 2006. Cost effectiveness of interferon A or peginterferon A with ribavirin for histologically mild chronic hepatitis C (Structured Abstract). Gut, 55.

GRIEVE, R., ROBERTS, J., WRIGHT, M., SWEETING, M., DEANGELIS, D., ROSENBERG, W., BASSENDINE, M., MAIN, J. & THOMAS, H. 2006. Cost effectiveness of interferon alpha or peginterferon alpha with ribavirin for histologically mild chronic hepatitis C. Gut, 55, 1332-8.

GRISHCHENKO, M., GRIEVE, R. D., SWEETING, M. J., DE ANGELIS, D., THOMSON, B. J., RYDER, S. D., IRVING, W. L. & TRENT, H. C. V. S. G. 2009. Cost-effectiveness of pegylated interferon and ribavirin for patients with chronic hepatitis C treated in routine clinical practice. Int J Technol Assess Health Care, 25, 171-80.

GUENKA, P., ASSENA, S. & MANOVA, M. 2009. Cost-effectiveness of peginterferon alfa-2b) in HCV infection: A Bulgarian Scenario. Archives of the Balkan Medical Union, 44, 215-219.

GURUSAMY, K. S., WILSON, E., KORETZ, R. L., ALLEN, V. B., DAVIDSON, B. R., BURROUGHS, A. K. & GLUUD, C. 2013. Is sustained virological response a marker of treatment efficacy in patients with chronic hepatitis C viral infection with no response or relapse to previous antiviral intervention? PLoS One, 8, e83313.

HAGAN, L. M., YANG, Z., EHTESHAMI, M. & SCHINAZI, R. F. 2013. All-oral, interferon-free treatment for chronic hepatitis C: cost-effectiveness analyses. J Viral Hepat, 20, 847-57.

HARTWELL, D., COOPER, K., FRAMPTON, G. K., BAXTER, L. & LOVEMAN, E. 2014. The clinical effectiveness and cost-effectiveness of peginterferon alfa and ribavirin for the treatment of chronic hepatitis C in children and young people: a systematic review and economic evaluation. Health Technol Assess, 18, i-xxii, 1-202.

HARTWELL, D., JONES, J., BAXTER, L. & SHEPHERD, J. 2011. Peginterferon alfa and ribavirin for chronic hepatitis C in patients eligible for shortened treatment, re-treatment or in HCV/HIV co-infection: a systematic review and economic evaluation. Health Technol Assess, 15, i-xii, 1-210.

HAYASHIDA, K., NAGASUE, I., FUKUDA, T. & GUNJI, A. 2002. The natural history model of hepatitis C virus infection and the economic evaluation of alpha interferon treatment. J Epidemiol, 12, 22-32.

HORNBERGER, J., FARCI, P., PRATI, D., ZEUZEM, S., GREEN, J. & PATEL, K. K. 2006. The economics of treating chronic hepatitis C patients with peginterferon alpha-2a (40 kDa) plus ribavirin presenting with persistently normal aminotransferase. J Viral Hepat, 13, 377-86.

HORNBERGER, J., TORRIANI, F. J., DIETERICH, D. T., BRAU, N., SULKOWSKI, M. S., TORRES, M. R., GREEN, J. & PATEL, K. 2006. Cost-effectiveness of peginterferon alfa-2a (40kDa) plus ribavirin in patients with HIV and hepatitis C virus co-infection. J Clin Virol, 36, 283-91.

HOSHIDA, Y., SHIRATORI, Y. & OMATA, M. 2002. Cost-effectiveness of adjuvant interferon therapy after surgical resection of Hepatitis C-related hepatocellular carcinoma. Liver, 22, 479-85.

HUANG, T., TU, K., SHYR, Y., WEI, C. C., XIE, L. & LI, Y. X. 2008. The prediction of interferon treatment effects based on time series microarray gene expression profiles. J Transl Med, 6, 44.

IANNAZZO, S., COLOMBATTO, P., RICCO, G., OLIVERI, F., BONINO, F. & BRUNETTO, M. R. 2015. A cost-effectiveness model to personalize antiviral therapy in naive patients with genotype 1 chronic hepatitis C. Dig Liver Dis, 47, 249-54.

IKEDA, K., KAWAMURA, Y., KOBAYASHI, M., FUKUSHIMA, T., SEZAKI, H., HOSAKA, T., AKUTA, N., SAITOH, S., SUZUKI, F., SUZUKI, Y., ARASE, Y. & KUMADA, H. 2014. Prevention of disease progression with anti-inflammatory therapy in patients with HCV-related cirrhosis: a Markov model. Oncology, 86, 295-302.

INNES, H., GOLDBERG, D., DUSHEIKO, G., HAYES, P., MILLS, P. R., DILLON, J. F., ASPINALL, E., BARCLAY, S. T. & HUTCHINSON, S. J. 2014. Patient-important benefits of clearing the hepatitis C virus through treatment: a simulation model. J Hepatol, 60, 1118-26.

ISHIDA, H., INOUE, Y., WONG, J. B. & OKITA, K. 2004. Cost-effectiveness of ribavirin plus interferon alpha-2b for either interferon relapsers or non-responders in chronic hepatitis C: a Japanese trial. Hepatol Res, 28, 125-136.

KIM, D. D., HUTTON, D. W., RAOUF, A. A., SALAMA, M., HABLAS, A., SEIFELDIN, I. A. & SOLIMAN, A. S. 2015. Cost-effectiveness model for hepatitis C screening and treatment: Implications for Egypt and other countries with high prevalence. Glob Public Health, 10, 296-317.

KIM, W. R., POTERUCHA, J. J., HERMANS, J. E., THERNEAU, T. M., DICKSON, E. R., EVANS, R. W. & GROSS, J. B., JR. 1997. Cost-effectiveness of 6 and 12 months of interferon-alpha therapy for chronic hepatitis C. Ann Intern Med, 127, 866-74.

KRAHN, M. D., JOHN-BAPTISTE, A., YI, Q., DORIA, A., REMIS, R. S., RITVO, P. & FRIEDMAN, S. 2005. Potential cost-effectiveness of a preventive hepatitis C vaccine in high risk and average risk populations in Canada. Vaccine, 23, 1549-58.

KUEHNE, F. C., BETHE, U., FREEDBERG, K. & GOLDIE, S. J. 2002. Treatment for hepatitis C virus in human immunodeficiency virus-infected patients: clinical benefits and cost-effectiveness. Arch Intern Med, 162, 2545-56.

KUROSAKI, M., HIRAMATSU, N., SAKAMOTO, M., SUZUKI, Y., IWASAKI, M., TAMORI, A., MATSUURA, K., KAKINUMA, S., SUGAUCHI, F., SAKAMOTO, N., NAKAGAWA, M., YATSUHASHI, H. & IZUMI, N. 2012. Age and total ribavirin dose are independent predictors of relapse after interferon therapy in chronic hepatitis C revealed by data mining analysis. Antivir Ther, 17, 35-43.

KUROSAKI, M., MATSUNAGA, K., HIRAYAMA, I., TANAKA, T., SATO, M., YASUI, Y., TAMAKI, N., HOSOKAWA, T., UEDA, K., TSUCHIYA, K., NAKANISHI, H., IKEDA, H., ITAKURA, J., TAKAHASHI, Y., ASAHINA, Y., HIGAKI, M., ENOMOTO, N. & IZUMI, N. 2010. A predictive model of response to peginterferon ribavirin in chronic hepatitis C using classification and regression tree analysis. Hepatol Res, 40, 251-60.

KUROSAKI, M., SAKAMOTO, N., IWASAKI, M., SAKAMOTO, M., SUZUKI, Y., HIRAMATSU, N., SUGAUCHI, F., TAMORI, A., NAKAGAWA, M. & IZUMI, N. 2011. Sequences in the interferon sensitivity-determining region and core region of hepatitis C virus impact pretreatment prediction of response to PEG-interferon plus ribavirin: data mining analysis. J Med Virol, 83, 445-52.

KUROSAKI, M., SAKAMOTO, N., IWASAKI, M., SAKAMOTO, M., SUZUKI, Y., HIRAMATSU, N., SUGAUCHI, F., YATSUHASHI, H. & IZUMI, N. 2011. Pretreatment prediction of response to peginterferon plus ribavirin therapy in genotype 1 chronic hepatitis C using data mining analysis. J Gastroenterol, 46, 401-9.

LIDGREN, M., HOLLANDER, A., WEILAND, O. & JONSSON, B. 2007. Productivity improvements in hepatitis C treatment: impact on efficacy, cost, cost-effectiveness and quality of life. Scand J Gastroenterol, 42, 867-77.

LIN, W. A., TARN, Y. H. & TANG, S. L. 2006. Cost-utility analysis of different peg-interferon alpha-2b plus ribavirin treatment strategies as initial therapy for naive Chinese patients with chronic hepatitis C. Aliment Pharmacol Ther, 24, 1483-93.

LOGGE, C., VETTORAZZI, E., FISCHER, L., NASHAN, B. & STERNECK, M. 2013. Cost-effectiveness analysis of antiviral treatment in liver transplant recipients with HCV infection. Transpl Int, 26, 527-34.

MALONE, D. C., TRAN, T. T. & POORDAD, F. F. 2005. Cost-efficacy analysis of peginterferon alfa-2b plus ribavirin compared with peginterferon alfa-2a plus ribavirin for the treatment of chronic hepatitis C. J Manag Care Pharm, 11, 687-94.

MANDRIK, O., KNIES, S., GOLUBOVSKA, O., DUDA, O., DUDAR, L., FEDORCHENKO, S., ZALISKA, O. & HANS SEVERENS, J. L. 2015. Cost comparison of treating chronic hepatitis C genotype one with pegylated interferons in Ukraine. Open Med (Wars), 10, 25-33.

MARTIN, N. K., PITCHER, A. B., VICKERMAN, P., VASSALL, A. & HICKMAN, M. 2011. Optimal control of hepatitis C antiviral treatment programme delivery for prevention amongst a population of injecting drug users. PLoS One, 6, e22309.

MARTIN, N. K., VICKERMAN, P., FOSTER, G. R., MINERS, A., HUTCHINSON, S. J., GOLDBERG, D. & HICKMAN, M. 2011. The cost-effectiveness of HCV antiviral treatment for injecting drug user populations. Gut, 60, A25.

MARTIN, N. K., VICKERMAN, P., MINERS, A., FOSTER, G. R., HUTCHINSON, S. J., GOLDBERG, D. J. & HICKMAN, M. 2012. Cost-effectiveness of hepatitis C virus antiviral treatment for injection drug user populations. Hepatology, 55, 49-57.

MCEWAN, P., KIM, R. & YUAN, Y. 2013. Assessing the cost utility of response-guided therapy in patients with chronic hepatitis C genotype 1 in the UK using the MONARCH model. Appl Health Econ Health Policy, 11, 53-63.

MEHRAZMAY, A., ALAVIAN, S. M., MORADI-LAKEH, M., MOKHTARI PAYAM, M., HASHEMI-MESHKINI, A., BEHNAVA, B., MIRI, S. M., KARIMI ELIZEE, P., TABATABAEE, S. V., KESHVARI, M. & BAGHERI LANKARANI, K. 2013. Cost-effectiveness analysis of adding low dose ribavirin to peginterferon alfa-2a for treatment of chronic hepatitis C infected thalassemia major patients in iran. Hepat Mon, 13, e10236.

MENNINI, F. S., MARCELLUSI, A., ANDREONI, M., GASBARRINI, A., SALOMONE, S. & CRAXI, A. 2014. Health policy model: long-term predictive results associated with the management of hepatitis C virus-induced diseases in Italy. Clinicoecon Outcomes Res, 6, 303-10.

MESSORI, A., FADDA, V., MARATEA, D. & TRIPPOLI, S. 2012. Effect of discounting on estimation of benefits determined by hepatitis C treatment. World J Gastroenterol, 18, 3032-4.

MINERS, A. H., MARTIN, N. K., GHOSH, A., HICKMAN, M. & VICKERMAN, P. 2014. Assessing the cost-effectiveness of finding cases of hepatitis C infection in UK migrant populations and the value of further research. J Viral Hepat, 21, 616-23.

MORIGUCHI, H., UEMURA, T., KOBAYASHI, M., CHUNG, R. T. & SATO, C. 2002. Management strategies using pharmacogenomics in patients with severe HCV-1b infection: a decision analysis. Hepatology, 36, 177-85.

MOSEGUI, G. B. G., VIANNA, C. M. D. M., RODRIGUES, M. P. D. S. & PEREZ, R. D. M. 2011. Alfapeguinterferon-2a e ribavirina versus alfapeguinterferon-2b e ribavirina: avaliação custo-efetividade e do impacto orçamentário do tratamento do genótipo 1 da hepatite C crônica. Physis: Revista de Saúde Coletiva, 21, 377-393.

MOURAD, A., DEUFFIC-BURBAN, S., GANNE-CARRIE, N., RENAUT-VANTROYS, T., ROSA, I., BOUVIER, A. M., LAUNOY, G., CATTAN, S., LOUVET, A., DHARANCY, S., TRINCHET, J. C., YAZDANPANAH, Y. & MATHURIN, P. 2014. Hepatocellular carcinoma screening in patients with compensated hepatitis C virus (HCV)-related cirrhosis aware of their HCV status improves survival: a modeling approach. Hepatology, 59, 1471-81.

MUNARI, L. M. & PICCIOTTO, A. 1996. Recombinant interferon alpha2b therapy of chronic hepatitis C in Italy: An economic analysis. FORUM - Trends in Experimental and Clinical Medicine, 6, 347-353.

NAKAMURA, J., KOBAYASHI, K., TOYABE, S., AOYAGI, Y. & AKAZAWA, K. 2007. The cost-effectiveness of the new protocol reflecting rapid virologic response to peginterferon alpha-2b and ribavirin for chronic hepatitis C. Eur J Gastroenterol Hepatol, 19, 733-9.

NAKAMURA, J., TOYABE, S. I., AOYAGI, Y. & AKAZAWA, K. 2008. Economic impact of extended treatment with peginterferon alpha-2a and ribavirin for slow hepatitis C virologic responders. J Viral Hepat, 15, 293-9.

OBACH, D., DEUFFIC-BURBAN, S., ESMAT, G., ANWAR, W. A., DEWEDAR, S., CANVA, V., COUSIEN, A., DOSS, W., MOSTAFA, A., POL, S., BUTI, M., SIEBERT, U., FONTANET, A., MOHAMED, M. K. & YAZDANPANAH, Y. 2014. Effectiveness and cost-effectiveness of immediate versus delayed treatment of hepatitis C virus-infected patients in a country with limited resources: the case of Egypt. Clin Infect Dis, 58, 1064-71.

PATEL, D., TERRAULT, N. A., YAO, F. Y., BASS, N. M. & LADABAUM, U. 2005. Cost-effectiveness of hepatocellular carcinoma surveillance in patients with hepatitis C virus-related cirrhosis. Clin Gastroenterol Hepatol, 3, 75-84.

POKORSKI, R. J. 2000. Long-term insured lives morbidity and mortality risk associated with chronic hepatitis C virus infection. J Insur Med, 32, 226-48.

POKORSKI, R. J. 2001. Long-term morbidity and mortality risk in Japanese insurance applicants with chronic hepatitis C virus infection. J Insur Med, 33, 12-36.

RAVASIO, R. 2008. [Cost-effectiveness of peginterferon alpha-2a plus ribavirin versus peginterferon alpha-2b plus ribavirin in the treatment of chronic hepatitis C in HIV-HCV co-infection]. Pharmacoeconomics Italian Research Articles, 10, 37-47.

RAVASIO, R., SACCHI, P., MAIOCCHI, L., PATRUNO, S., BRUNO, R. & FILICE, G. 2005. Costo efficacia di peginterferone α-2a + ribavirina verso peginterferone α-2b + ribavirina nel trattamento dell’epatite cronica di tipo C in pazienti non pretrattati. PharmacoEconomics Italian Research Articles, 7, 207-218.

ROBERTS, J. A. 1999. The economic aspects of hepatitis C-- implications for haemophilia. Haemophilia, 5, 402-9.

RODRIGUES, M. P., VIANNA, C. M., MOSEGUI, G. B., COSTA E SILVA, F. V., PEREGRINO, A. A. & JARDIM, F. N. 2013. [Cost-effectiveness of hepatitis C treatment in slow virologic responders coinfected with HIV]. Cad Saude Publica, 29 Suppl 1, S146-58.

SAAB, S., HUNT, D. R., STONE, M. A., MCCLUNE, A. & TONG, M. J. 2010. Timing of hepatitis C antiviral therapy in patients with advanced liver disease: a decision analysis model. Liver Transpl, 16, 748-59.

SACKS-DAVIS, R., MCBRYDE, E., GREBELY, J., HELLARD, M. & VICKERMAN, P. 2015. Many hepatitis C reinfections that spontaneously clear may be undetected: Markov-chain Monte Carlo analysis of observational study data. J R Soc Interface, 12, 20141197.

SAGMEISTER, M., WONG, J. B., MULLHAUPT, B. & RENNER, E. L. 2001. A pragmatic and cost-effective strategy of a combination therapy of interferon alpha-2b and ribavirin for the treatment of chronic hepatitis C. Eur J Gastroenterol Hepatol, 13, 483-8.

SALOMON, J. A., WEINSTEIN, M. C., HAMMITT, J. K. & GOLDIE, S. J. 2003. Cost-effectiveness of treatment for chronic hepatitis C infection in an evolving patient population. JAMA, 290, 228-37.

SAN MIGUEL, R., MAR, J., CABASES, J. M., GUILLEN-GRIMA, F. & BUTI, M. 2003. Cost-effectiveness analysis of therapeutic strategies for patients with chronic hepatitis C previously not responding to interferon. Aliment Pharmacol Ther, 17, 765-73.

SENNFALT, K., REICHARD, O., HULTKRANTZ, R., WONG, J. B. & JONSSON, D. 2001. Cost-effectiveness of interferon alfa-2b with and without ribavirin as therapy for chronic hepatitis C in Sweden. Scand J Gastroenterol, 36, 870-6.

SHEPHERD, J., BRODIN, H., CAVE, C., WAUGH, N., PRICE, A. & GABBAY, J. 2004. Pegylated interferon alpha-2a and -2b in combination with ribavirin in the treatment of chronic hepatitis C: a systematic review and economic evaluation. Health Technol Assess, 8, iii-iv, 1-125.

SHEPHERD, J., BRODIN, H. F., CAVE, C. B., WAUGH, N. R., PRICE, A. & GABBAY, J. 2005. Clinical- and cost-effectiveness of pegylated interferon alfa in the treatment of chronic hepatitis C: a systematic review and economic evaluation. Int J Technol Assess Health Care, 21, 47-54.

SHEPHERD, J. & JONES, J. 2007. A systematic review of the cost-effectiveness of peginterferon alfa-2b in the treatment of chronic hepatitis C. Expert Rev Pharmacoecon Outcomes Res, 7, 577-95.

SHEPHERD, J., JONES, J., HARTWELL, D., DAVIDSON, P., PRICE, A. & WAUGH, N. 2007. Interferon alpha (pegylated and non-pegylated) and ribavirin for the treatment of mild chronic hepatitis C: a systematic review and economic evaluation. Health Technol Assess, 11, 1-205, iii.

SHEPHERD, J., WAUGH, N. & HEWITSON, P. 2000. Combination therapy (interferon alfa and ribavirin) in the treatment of chronic hepatitis C: a rapid and systematic review. Health Technol Assess, 4, 1-67.

SHIELL, A., BRIGGS, A. & FARRELL, G. C. 1994. The cost effectiveness of alpha interferon in the treatment of chronic active hepatitis C. Med J Aust, 160, 268-72.

SHIELL, A., BROWN, S. & FARRELL, G. C. 1999. Hepatitis C: an economic evaluation of extended treatment with interferon. Med J Aust, 171, 189-93.

SIEBERT, U. & SROCZYNSKI, G. 2003. Antiviral therapy for patients with chronic hepatitis C in Germany - evaluation of effectiveness and cost effectiveness of initial combination therapy with interferon / peginterferon plus ribavarin (structured abstract) HTA-3200500570. York (UK): CRD YORK HTA Database.

SIEBERT, U., SROCZYNSKI, G., AIDELSBURGER, P., ROSSOL, S., WASEM, J., MANNS, M. P., MCHUTCHISON, J. G. & WONG, J. B. 2009. Clinical effectiveness and cost effectiveness of tailoring chronic hepatitis C treatment with peginterferon alpha-2b plus ribavirin to HCV genotype and early viral response: a decision analysis based on German guidelines. Pharmacoeconomics, 27, 341-54.

SIEBERT, U., SROCZYNSKI, G., GERMAN HEPATITIS, C. M. G. & C, H. T. A. E. P. O. H. 2003. Antiviral combination therapy with interferon/peginterferon plus ribavirin for patients with chronic hepatitis C in Germany: a health technology assessment commissioned by the German Agency for Health Technology Assessment. Ger Med Sci, 1, Doc07.

SIEBERT, U., SROCZYNSKI, G., GERMAN HEPATITIS, C. M. G. G. & C, H. T. A. E. P. O. H. 2005. Effectiveness and cost-effectiveness of initial combination therapy with interferon/peginterferon plus ribavirin in patients with chronic hepatitis C in Germany: a health technology assessment commissioned by the German Federal Ministry of Health and Social Security. Int J Technol Assess Health Care, 21, 55-65.

SIEBERT, U., SROCZYNSKI, G., ROSSOL, S., WASEM, J., RAVENS-SIEBERER, U., KURTH, B. M., MANNS, M. P., MCHUTCHISON, J. G., WONG, J. B., GERMAN HEPATITIS, C. M. G. & INTERNATIONAL HEPATITIS INTERVENTIONAL THERAPY, G. 2003. Cost effectiveness of peginterferon alpha-2b plus ribavirin versus interferon alpha-2b plus ribavirin for initial treatment of chronic hepatitis C. Gut, 52, 425-32.

SIMON, K., GLADYSZ, A., ROTTER, K., RYMER, W., SMOLINSKI, P., INGLOT, M. & WLADYSIUK-BLICHARZ, M. 2006. Cost effectiveness of replacing recombinated interferon alpha-2b with its pegylated form in combination with ribavirin for the therapy of chronic HCV infection in Poland. Advanced in Experimental Medicine.

SINHA, M. & DAS, A. 2000. Cost effectiveness analysis of different strategies of management of chronic hepatitis C infection in children. Pediatr Infect Dis J, 19, 23-30.

SNOECK, E., HADZIYANNIS, S. J., PUOTI, C., SWAIN, M. G., BERG, T., MARCELLIN, P., ZARSKI, J. P., JORGA, K. & ZEUZEM, S. 2008. Predicting efficacy and safety outcomes in patients with hepatitis C virus genotype 1 and persistently 'normal' alanine aminotransferase levels treated with peginterferon alpha-2a (40KD) plus ribavirin. Liver Int, 28, 61-71.

STEIN, K., ROSENBERG, W. & WONG, J. 2002. Cost effectiveness of combination therapy for hepatitis C: a decision analytic model. Gut, 50, 253-8.

SULLIVAN, S. D., CRAXI, A., ALBERTI, A., GIULIANI, G., DE CARLI, C., WINTFELD, N., PATEL, K. K. & GREEN, J. 2004. Cost effectiveness of peginterferon alpha-2a plus ribavirin versus interferon alpha-2b plus ribavirin as initial therapy for treatment-naive chronic hepatitis C. Pharmacoeconomics, 22, 257-65.

SULLIVAN, S. D., CRAXI, A., ALBERTI, A., GIULIANI, G., DE CARLI, C., WINTFELD, N., PATEL, K. K. & GREEN, J. 2004. Cost effectiveness of peginterferon alpha-2a plus ribavirin versus interferon alpha-2b plus ribavirin as initial therapy for treatment-naive chronic hepatitis C. Pharmacoeconomics, 22, 257-65.

SULLIVAN, S. D., CRAXI, A., ALBERTI, A., GIULIANI, G., DE CARLI, C., WINTFELD, N., PATEL, K. K. & GREEN, J. 2004. Cost effectiveness of peginterferon alpha-2b plus ribavirin versus interferon alpha-2b plus ribavirin as initial therapy for treatment-naive chronic hepatitis C. Pharmacoeconomics Italian Research Articles, 6, 105-114.

SULLIVAN, S. D., JENSEN, D. M., BERNSTEIN, D. E., HASSANEIN, T. I., FOSTER, G. R., LEE, S. S., CHEINQUER, H., CRAXI, A., COOKSLEY, G., KLASKALA, W., PETTIT, K., PATEL, K. K. & GREEN, J. 2004. Cost-effectiveness of combination peginterferon alpha-2a and ribavirin compared with interferon alpha-2b and ribavirin in patients with chronic hepatitis C. Am J Gastroenterol, 99, 1490-6.

SWEETING, M. J., DE ANGELIS, D., NEAL, K. R., RAMSAY, M. E., IRVING, W. L., WRIGHT, M., BRANT, L., HARRIS, H. E., TRENT, H. C. V. S. G. & GROUP, H. C. V. N. R. S. 2006. Estimated progression rates in three United Kingdom hepatitis C cohorts differed according to method of recruitment. J Clin Epidemiol, 59, 144-52.

SWEETING, M. J., FAREWELL, V. T. & DE ANGELIS, D. 2010. Multi-state Markov models for disease progression in the presence of informative examination times: an application to hepatitis C. Stat Med, 29, 1161-74.

TAKEDA, A., JONES, J., SHEPHERD, J., DAVIDSON, P. & PRICE, A. 2007. A systematic review and economic evaluation of adefovir dipivoxil and pegylated interferon-alpha-2a for the treatment of chronic hepatitis B. J Viral Hepat, 14, 75-88.

TERRAULT, N. A., IM, K., BOYLAN, R., BACCHETTI, P., KLEINER, D. E., FONTANA, R. J., HOOFNAGLE, J. H., BELLE, S. H. & GROUP, V.-C. S. 2008. Fibrosis progression in African Americans and Caucasian Americans with chronic hepatitis C. Clin Gastroenterol Hepatol, 6, 1403-11.

THOMPSON COON, J., ROGERS, G., HEWSON, P., WRIGHT, D., ANDERSON, R., CRAMP, M., JACKSON, S., RYDER, S., PRICE, A. & STEIN, K. 2007. Surveillance of cirrhosis for hepatocellular carcinoma: systematic review and economic analysis. Health Technol Assess, 11, 1-206.

TURNBULL, C. & SUGANO, D. 1994. Cost effectiveness of interferon in chronic active hepatitis C. Med J Aust, 161, 169; author reply 170.

TURNES, J., ROMERO-GOMEZ, M., PLANAS, R., SOLA, R., GARCIA-SAMANIEGO, J., DIAGO, M., CRESPO, J., CALLEJA, J. L., RUBIO-TERRES, C. & VENTAYOL, P. 2013. Pharmacoeconomic analysis of the treatment of chronic hepatitis C with peginterferon alfa-2a or peginterferon alfa-2b plus ribavirin in Spain. Gastroenterol Hepatol, 36, 555-64.

VACHON, M. L. & DIETERICH, D. T. 2012. A great time to invest in baby Boomer's hepatitis C! Hepatology, 56, 1575-7.

VAN LEEUWEN, D. J. 1999. Cost-effectiveness of interferon treatment for hepatitis C. JAMA, 281, 2083-4.

VISCONTI, A. J., DOYLE, J. S., WEIR, A., SHIELL, A. M. & HELLARD, M. E. 2013. Assessing the cost-effectiveness of treating chronic hepatitis C virus in people who inject drugs in Australia. J Gastroenterol Hepatol, 28, 707-16.

VOLK, M. L., TOCCO, R., SAINI, S. & LOK, A. S. 2009. Public health impact of antiviral therapy for hepatitis C in the United States. Hepatology, 50, 1750-5.

WERB, D., WOOD, E., KERR, T., HERSHFIELD, N., PALMER, R. W. & REMIS, R. S. 2011. Treatment costs of hepatitis C infection among injection drug users in Canada, 2006-2026. Int J Drug Policy, 22, 70-6.

WONG, J. & NEVENS, F. 2002. Cost-effectiveness of peginterferon alfa-2b plus ribavirin compared to interferon alfa-2b plus ribavirin as initial treatment of chronic hepatitis C in Belgium. Acta Gastroenterol Belg, 65, 110-11.

WONG, J. B. 1998. Interferon treatment for chronic hepatitis B or C infection: costs and effectiveness. Acta Gastroenterol Belg, 61, 238-42.

WONG, J. B. 1999. Cost-effectiveness of treatments for chronic hepatitis C. Am J Med, 107, 74S-78S.

WONG, J. B., BENNETT, W. G., KOFF, R. S. & PAUKER, S. G. 1998. Pretreatment evaluation of chronic hepatitis C: risks, benefits, and costs. JAMA, 280, 2088-93.

WONG, J. B., DAVIS, G. L. & PAUKER, S. G. 2000. Cost effectiveness of ribavirin/interferon alfa-2b after interferon relapse in chronic hepatitis C. Am J Med, 108, 366-73.

WONG, J. B. & KOFF, R. S. 2000. Watchful waiting with periodic liver biopsy versus immediate empirical therapy for histologically mild chronic hepatitis C. A cost-effectiveness analysis. Ann Intern Med, 133, 665-75.

WONG, J. B., POYNARD, T., LING, M. H., ALBRECHT, J. K. & PAUKER, S. G. 2000. Cost-effectiveness of 24 or 48 weeks of interferon alpha-2b alone or with ribavirin as initial treatment of chronic hepatitis C. International Hepatitis Interventional Therapy Group. Am J Gastroenterol, 95, 1524-30.

YEH, W. S., ARMSTRONG, E. P., SKREPNEK, G. H. & MALONE, D. C. 2007. Peginterferon alfa-2a versus peginterferon alfa-2b as initial treatment of hepatitis C virus infection: a cost-utility analysis from the perspective of the Veterans Affairs Health Care System. Pharmacotherapy, 27, 813-24.

YOUNOSSI, Z. M., SINGER, M. E., MCHUTCHISON, J. G. & SHERMOCK, K. M. 1999. Cost effectiveness of interferon alpha2b combined with ribavirin for the treatment of chronic hepatitis C. Hepatology, 30, 1318-24.

YOUNOSSI, Z. M., SINGER, M. E., MIR, H. M., HENRY, L. & HUNT, S. 2014. Impact of interferon free regimens on clinical and cost outcomes for chronic hepatitis C genotype 1 patients. J Hepatol, 60, 530-7.

**Reason for exclusion, outcome (n=4)**

DEUFFIC-BURBAN, S., CASTEL, H., WIEGAND, J., MANNS, M. P., WEDEMEYER, H., MATHURIN, P. & YAZDANPANAH, Y. 2012. Immediate vs. delayed treatment in patients with acute hepatitis C based on IL28B polymorphism: a model-based analysis. J Hepatol, 57, 260-6.

ECKMAN, M. H., TALAL, A. H., GORDON, S. C., SCHIFF, E. & SHERMAN, K. E. 2013. Cost-effectiveness of screening for chronic hepatitis C infection in the United States. Clin Infect Dis, 56, 1382-93.

LIN, O. S., KEEFFE, E. B., SANDERS, G. D. & OWENS, D. K. 2004. Cost-effectiveness of screening for hepatocellular carcinoma in patients with cirrhosis due to chronic hepatitis C. Aliment Pharmacol Ther, 19, 1159-72.

MCEWAN, P., WARD, T., BENNETT, H., KALSEKAR, A., WEBSTER, S., BRENNER, M. & YUAN, Y. 2015. Estimating the clinical and economic benefit associated with incremental improvements in sustained virologic response in chronic hepatitis C. PLoS One, 10, e0117334.

**Reason for exclusion, study design (n=32)**

CHONG, C. A., GULAMHUSSEIN, A., HEATHCOTE, E. J., LILLY, L., SHERMAN, M., NAGLIE, G. & KRAHN, M. 2003. Health-state utilities and quality of life in hepatitis C patients. Am J Gastroenterol, 98, 630-8.

DORE, G. J., FREEMAN, A. J., LAW, M. & KALDOR, J. M. 2002. Is severe liver disease a common outcome for people with chronic hepatitis C? J Gastroenterol Hepatol, 17, 423-30.

DORE, G. J. & THEIN, H. H. 2003. Cost-effectiveness of treatment for chronic hepatitis C infection. JAMA, 290, 1993; author reply 1994.

GELLAD, Z. F., REED, S. D. & MUIR, A. J. 2012. Economic evaluation of direct-acting antiviral therapy in chronic hepatitis C. Antivir Ther, 17, 1189-99.

HAGAN, L. 2014. Cost-effectiveness and access to care in the treatment of hepatitis C virus infection. Gastroenterol Hepatol (N Y), 10, 259-61.

HARTWELL, D., JONES, J., BAXTER, L. & SHEPHERD, J. 2012. Shortened peginterferon and ribavirin treatment for chronic hepatitis C. Int J Technol Assess Health Care, 28, 398-406.

HASHEM, A. M., RASMY, M. E., WAHBA, K. M. & SHAKER, O. G. 2012. Single stage and multistage classification models for the prediction of liver fibrosis degree in patients with chronic hepatitis C infection. Comput Methods Programs Biomed, 105, 194-209.

HENLEY, E. 1999. Cost-effectiveness of interferon treatment for hepatitis C. JAMA, 281, 2083; author reply 2084.

JOHN-BAPTISTE, A., YEUNG, M. W., LEUNG, V., VAN DER VELDE, G. & KRAHN, M. 2012. Cost effectiveness of hepatitis C-related interventions targeting substance users and other high-risk groups: a systematic review. Pharmacoeconomics, 30, 1015-34.

KABIRI, M., JAZWINSKI, A. B., ROBERTS, M. S., SCHAEFER, A. J. & CHHATWAL, J. 2014. The changing burden of hepatitis C virus infection in the United States: model-based predictions. Ann Intern Med, 161, 170-80.

KAWAMURA, Y., TAKASAKI, S. & MIZOKAMI, M. 2012. Using decision tree learning to predict the responsiveness of hepatitis C patients to drug treatment. FEBS Open Bio, 2, 98-102.

KELTCH, B., LIN, Y. & BAYRAK, C. 2014. Comparison of AI techniques for prediction of liver fibrosis in hepatitis patients. J Med Syst, 38, 60.

KHATRI, N., LATHER, V. & MADAN, A. K. 2015. Diverse models for anti-HIV activity of purine nucleoside analogs. Chem Cent J, 9, 29.

KIM, W. R., POTERUCHA, J. J. & GROSS, J. B., JR. 2000. Cost-effectiveness of interferon alfa 2b and ribavirin in the treatment of chronic hepatitis C. Hepatology, 31, 807-8.

KRAHN, M., WONG, J. B., HEATHCOTE, J., SCULLY, L. & SEEFF, L. 2004. Estimating the prognosis of hepatitis C patients infected by transfusion in Canada between 1986 and 1990. Med Decis Making, 24, 20-9.

KUROSAKI, M., HIRAMATSU, N., SAKAMOTO, M., SUZUKI, Y., IWASAKI, M., TAMORI, A., MATSUURA, K., KAKINUMA, S., SUGAUCHI, F., SAKAMOTO, N., NAKAGAWA, M. & IZUMI, N. 2012. Data mining model using simple and readily available factors could identify patients at high risk for hepatocellular carcinoma in chronic hepatitis C. J Hepatol, 56, 602-8.

LOUBIERE, S., ROTILY, M. & MOATTI, J. P. 2000. [Medico-economic assessment of the therapeutic management of patients with hepatitis C]. Gastroenterol Clin Biol, 24, 1047-51.

LOUBIERE, S., ROTILY, M. & MOATTI, J. P. 2002. Appraisal of economic evaluations of treatments and screening for hepatitis C. Med Sci (Paris), 18, 325-333.

MANNS, M. P. 2004. Adherence to combination therapy: influence on sustained virologic response and economic impact. Gastroenterol Clin North Am, 33, S11-24.

MARCELLUSI, A., VITI, R., CAPONE, A. & MENNINI, F. S. 2014. [Cost of illness probabilistic methodology to assess direct and indirect costs of HCV-related disease in Italy]. Pharmacoeconomics Italian Research Articles, 16, 23.

MARTIN, N. K., VICKERMAN, P., MINERS, A. & HICKMAN, M. 2013. How cost-effective is hepatitis C virus treatment for people who inject drugs? J Gastroenterol Hepatol, 28, 590-2.

MYERS, R. P., KRAJDEN, M., BILODEAU, M., KAITA, K., MAROTTA, P., PELTEKIAN, K., RAMJI, A., ESTES, C., RAZAVI, H. & SHERMAN, M. 2014. Burden of disease and cost of chronic hepatitis C infection in Canada. Can J Gastroenterol Hepatol, 28, 243-50.

NEOH, C. F. & KONG, D. C. 2014. The cost-effectiveness of boceprevir for hepatitis C. Expert Rev Pharmacoecon Outcomes Res, 14, 319-34.

ORLEWSKA, E. 2004. Cost-effectiveness of pegylated IFN-alpha2b and -2a and ribavirin for chronic hepatitis C treatment. Expert Rev Pharmacoecon Outcomes Res, 4, 495-504.

RUGER, J. P., ABDALLAH, A. B., NG, N. Y., LUEKENS, C. & COTTLER, L. 2014. Cost-effectiveness of interventions to prevent HIV and STDs among women: a randomized controlled trial. AIDS Behav, 18, 1913-23.

SHERMOCK, K. M., TEMPLE, M. E. & YOUNOSSI, Z. M. 2002. The cost-effectiveness of treating chronic hepatitis C. Drug Benefit Trends.

SPAULDING, A. S., KIM, A. Y., HARZKE, A. J., SULLIVAN, J. C., LINAS, B. P., BREWER, A., DICKERT, J., MCGOVERN, B. H., STRICK, L. B., TRESTMAN, R. & FERGUSON, W. J. 2013. Impact of new therapeutics for hepatitis C virus infection in incarcerated populations. Top Antivir Med, 21, 27-35.

TANAKA, J., KUMADA, H., IKEDA, K., CHAYAMA, K., MIZUI, M., HINO, K., KATAYAMA, K., KUMAGAI, J., KOMIYA, Y., MIYAKAWA, Y. & YOSHIZAWA, H. 2003. Natural histories of hepatitis C virus infection in men and women simulated by the Markov model. J Med Virol, 70, 378-86.

THORLUND, K., DRUYTS, E., EL KHOURY, A. C. & MILLS, E. J. 2012. Budget impact analysis of boceprevir and telaprevir for the treatment of hepatitis C genotype 1 infection. Clinicoecon Outcomes Res, 4, 349-59.

TRAYNOR, K. 2014. Effectiveness, costs weigh on HCV treatment decisions. Am J Health Syst Pharm, 71, 1156-7.

TSOCHATZIS, E. A., CROSSAN, C. & LONGWORTH, L. 2014. Cost-effectiveness of upcoming treatments for hepatitis C: we need to get the models right. J Hepatol, 61, 453-4.

YI, Q., WANG, P. P. & KRAHN, M. 2004. Improving the accuracy of long-term prognostic estimates in hepatitis C virus infection. J Viral Hepat, 11, 166-74.

**Reason for exclusion, abstract only (n=121)**

AHMED, A., GORDON, S. C., SAAB, S. & YOUNOSSI, Z. 2014. Evaluation of the health outcomes for ledipasvir/ sofosbuvir in early vs. Delayed treatment according to fibrosis stage of patients with chronic hepatitis C virus (HCV) genotype 1 infection: Results from a decision-analytic markov model. Hepatology, 60, 1041A.

ALMADIYEVA, A., KOSTYUK, A. & NURGOZHIN, T. 2013. Compara tive effectiveness of triple therapy versus dual therapy for chronic hepatitisc virus infection in Kazakhstan. Value in Health, 16, A345.

AREIAS, J., BRANCO, T., CALINAS, F., CARVALHO, A., MACEDO, G., MANATA, M. J., MATOS, L., RODRIGUES, B., VELOSA, J., PEREIRA, C., SANCHES, M. & RUBIO-TERRES, C. 2011. Treatment of chronic hepatitis C patients with peginterferon alfa-2a or peginterferon alfa-2b: A cost-effectiveness analysis for the Portuguese NHS setting. Value in Health, 14, A394-A395.

ATHANASAKIS, K., KARAMPLI, E., RETSA, M. P., THEODOROPOULOU, T. & KYRIOPOULOS, J. 2013. Cost effectiveness analysis of boceprevir (BOC) added to pegifn/ ribavirin (p/r) versus pegifn/ribavirin (current standard of care) for the treatment of patients with genotype 1 chronic hepatitisc in Greece. Value in Health, 16, A352.

BARROS, F. M. R., CHEINQUER, H., BORGES, L. G. & SANTOS, E. 2010. Cost-effectiveness analysis of treatment with peginterferon-alfa-2a versus peginterferon-alfa-2b for patients with genotype 1 chronic hepatitis C under the public payer perspective in Brazil. Value in Health, 13, A72.

BARROS, F. M. R., CHEINQUER, H., BORGES, L. G. & SANTOS, E. 2010. Cost-effectiveness analysis of treatment with peginterferon-alfa-2a versus peginterferon-alfa-2b for patients with genotypes 2/3 chronic hepatitis C under the public payer perspective in Brazil. Value in Health, 13, A71-A72.

BECKER, B., CHHATWAL, J., FERRANTE, S., ELBASHA, E. H. & KROBOT, K. J. 2012. Cost-effectiveness of boceprevir-based treatment of chronic genotype 1 hepatitis C virus (HCV) infection from the perspective of the german statutory health insurance (SHI). Value in Health, 15, A392.

BECKER, B., CHHATWAL, J., FERRANTE, S., ELBASHA, E. H. & KROBOT, K. J. 2012. Projecting the clinical impact of treating hepatitis c genotype 1 infection with boceprevir in germany. Value in Health, 15, A387.

BLATT, C. R., STORB, B. H., MUHLBERGER, N., WURM, J., FARIAS, M. R. & SIEBERT, U. 2011. Chronic hepatitis C treatment for genotype 2 or 3: Costeffectiveness analysis of PEG as first line treatment with the Brazilian protocol. Value in Health, 14, A540.

BROGAN, A., MILLER, J., TALBIRD, S., THOMPSON, J. & DENIZ, B. 2011. Cost-effectiveness assessment of telaprevir combination treatment compared to pegylated-interferon+ribavirin alone in the management of chronic hepatitis c in treatment-naive patients. American Journal of Gastroenterology, 106, S410.

BROGAN, A., TALBIRD, S. E., THOMPSON, J. R., MILLER, J. D., GEORGE, S. & HVIDSTEN, K. 2012. Cost-effectiveness of first-line versus second-line telaprevir combination treatment in the management of chronic hepatitis c virus infection for treatment-naive patients with IL28B genotype CC. Gastroenterology, 142, S173.

BROGAN, A. J., MILLER, J. D., TALBIRD, S. E., THOMPSON, J. R. & DENIZ, B. 2011. Long-term clinical value of telaprevir for treatment of treatment-naive and treatment-experienced patients with hepatitis C virus (HCV) infection: Projections using decision-analytic modeling. Gastroenterology, 140, S947.

BUTI, M., GROS, B., OYAGUEZ, I., ANDRADE, R. J., SERRA, M. A., TURNES, J. & CASADO, M. A. 2013. Cost-effectiveness analysis of telaprevir triple therapy for treatment-naive patients with chronic hepatitis C based on the combined efficacy data of the ADVANCE and OPTIMIZE studies. Hepatology, 58, 1133A.

CANAVAN, C., COREY, K. & HUR, C. 2013. Defining cirrhosis with fibroscan for entry to hepatocellular carcinoma surveillance in chronic hepatitis C: A UK cost effectiveness analysis. Gut, 62, A35.

CARLOS, F. & DE, H. 2010. Cost-effectiveness of peginterferon alpha-2a plus ribavirin for treating cronic hepatitis c virus infection compared with no treatment in Mexico. Value in Health, 13, A439.

CARLOS, F. & DEHESA 2010. Cost-effectiveness of peginterferon alpha-2a versus peginterferon alpha-2b for treatment of chronic hepattis c infection in Mexico. Value in Health, 13, A439.

CHAN, K., LAI, M. N., GROESSL, E. J., HANCHATE, A., HERNANDEZ, L., WONG, J. B., CLARK, J. A., ASCH, S., GIFFORD, A. L. & HO, S. 2011. Potential costs associated with new direct acting antiviral (DAAS) therapy for untreated chronic hepatitis C genotype 1 infection in the veterans health administration. Value in Health, 14, A269-A270.

CHAN, K., LAI, M. N., GROESSL, E. J., HANCHATE, A. D., HERNANDEZ, L., WONG, J. B., CLARK, J., ASCH, S., GIFFORD, A. L. & HO, S. B. 2011. Long term clinical impact of direct antiviral agent (DAA) therapy for untreated chronic hepatitis C genotype 1 infection in the veterans health administration. Hepatology, 54, 437A-438A.

CHEN, W. & WEI, L. 2011. Cost-effectiveness analysis of peg-interferon alpha-2a plus ribavirin versus conventional interferon alpha-2a plus ribavirin for the treatment of chronic hepatitis C in China. Value in Health, 14, A280.

CHHATWAL, J., CHOPRA, K. B., ROBERTS, M. S. & DUNN, M. A. 2013. Framework for determining the cost-effectiveness of new antiviral agents for hepatitis c. Hepatology, 58, 386A-387A.

CHHATWAL, J., DUNN, M. A., ROBERTS, M. S. & CHOPRA, K. B. 2012. Prioritization of hepatitis C patients for treatment with direct acting antiviral agents. Hepatology, 56, 268A-269A.

CHHATWAL, J., FERRANTE, S. A., DASBACH, E. J., EL KHOURY, A., BRASS, C. A., BURROUGHS, M., BACON, B. R., ESTEBAN, R. & ELBASHA, E. 2011. Cost-effectiveness of boceprevir use in patients with chronic hepatitis C genotype-1 who failed prior treatment with peginterferon/ ribavirin. Hepatology, 54, 801A-802A.

CHHATWAL, J., FERRANTE, S. A., DASBACH, E. J., EL KHOURY, A., BURROUGHS, M., BACON, B., ESTEBAN-MUR, R. & BRASS, C. 2011. Projecting the long-term clinical impact of Boceprevir in patients with chronic hepatitis C genotype-1 who failed prior treatment with Peginterferon/ribavirin. Journal of Hepatology, 54, S164.

CHHATWAL, J., FERRANTE, S. A., DASBACH, E. J., EL KHOURY, A., BURROUGHS, M., BACON, B. R., ESTEBAN, R. & BRASS, C. A. 2011. Projecting the long-term clinical impact of boceprevir in patients with chronic hepatitis C genotype-1 who failed prior treatment with peginterferon/ribavirin. Gastroenterology, 140, S947.

CHHATWAL, J., KANWAL, F., ROBERTS, M. S. & DUNN, M. A. 2014. The economic impact of sofosbuvir-and simeprevir-based HCV treatment in the United States. Hepatology, 60, 255A-256A.

CHHATWAL, J., LUNDBERG, J., FERRANTE, S., EL KHOURHY, A. C., OKSANEN, A. & ELBASHA, E. H. 2012. Cost-effectiveness of boceprevir in the treatment of chronic Hepatitis c genotype 1 in Sweden. Journal of Hepatology, 56, S386-S387.

CHIDI, A. P., ROGAL, S. S., BRYCE, C. L., FINE, M. J., GOOD, C. B., MYASKOVSKY, L., RUSTGI, V. K., TSUNG, A. & SMITH, K. J. 2014. Cost-effectiveness of novel hepatitis C drug regimens among treatment-experienced U.S. veterans. Hepatology, 60, 234A.

COOPER, K., BAXTER, L., LOVEMAN, E., HARTWELL, D. & FRAMPTON, G. 2013. The cost effectiveness of peginterferon alfa and ribavirin for the treatment of hepatitisc in children and young people. Value in Health, 16, A499.

CORTESI, P. A., CIACCIO, A., ROTA, M., DE SALVIA, S., OKOLICSANYI, S., VINCI, M., BELLI, L. S., LIM, J. K., MANTOVANI, L. G. & STRAZZABOSCO, M. 2014. Management of chronic hepatitis C (CHC) genotype 1 treatment-naive patients in an era of rising opportunities and costs: A cost-effectiveness analysis. Digestive and Liver Disease, 46, e11.

CORTESI, P. A., CIACCIO, A., ROTA, M., DE SALVIA, S., VINCI, M., BELLI, L. S., LIM, J. K., MANTOVANI, L. G. & STRAZZABOSCO, M. 2014. Management of chronic hepatitis C (CHC) genotype 1 treatment-naive patients in an era of rising opportunities and costs-a cost-effectiveness analysis of treatment options. Journal of Hepatology, 60, S493.

CURE, S., BIANIC, F., CAWSTON, H., CAWSTON, L. & ZHANG, F. 2010. Impact of sustained virological response (SVR) on life expectancy and quality-adjusted life-years (QALYs) in chronic hepatitis C (CHC) patients. Journal of Hepatology, 52, S421.

CURE, S., BIANIC, F., CAWSTON, H., DARTOIS, L. & ZHANG, H. 2010. Estimated impact of sustained virological response (SVR) on life expectancy, quality-adjusted life-years (QALYS) and lifetime costs in chronic hepatitis c (CHC) patients. Value in Health, 13, A430.

CURE, S., BIANIC, F., DARTOIS, L., CAWSTON, H. & ZHANG, H. 2010. Impact of sustained virological response (SVR) on life expectancy and quality-adjusted life-years (QALYS) in chronic hepatitis C (CHC) patients. Value in Health, 13, A187.

CURE, S., CURTIS, S., BIANIC, F., GAVART, S., DEARDEN, L., FLEISCHMANN, J., OUWENS, M. & LEE, S. 2012. The cost-effectiveness of telaprevir (TVR) in combination with pegylated interferon-alfa and ribavirin (PR) for the treatment of genotype 1(G1) chronic Hepatitis C patients. Value in Health, 15, A241-A242.

CURE, S. & GUERRA, I. 2014. Cost-effectiveness and long-term outcomes of Sovaldi (sofosbuvir) for the treatment of chronic hepatitis c infected (HCV) patients from a Swedish societal perspective. Value in Health, 17, A675.

CURRY, M. P. & BLEIBEL, W. 2012. Update on the treatment of hepatitis C genotype 1. Journal of Clinical Outcomes Management, 19, 422-431.

CURTIS, S., CURE, S., BIANIC, F., GAVART, S., DEARDEN, L., FLEISCHMANN, J., OUWENS, M. & LEE, S. 2012. The cost-effectiveness of telaprevir (TVR) in Combination with pegylated interferon-alfa and ribavirin (PR) for the treatment of Genotype 1(G1) chronic hepatitis c patients: A post-hoc analysis of IL-28B subgroup. Value in Health, 15, A244-A245.

CURTIS, S., CURE, S., GAVART, S., DEARDEN, L., FLEISCHMANN, J., OUWENS, M. & LEE, S. 2012. The cost-effectiveness of telaprevir (TVR) in combination with pegylated interferon-alfa and ribavirin (PR) for the treatment of genotype 1 chronic hepatitis C patients. Journal of Hepatology, 56, S434.

DAN, Y. Y., FERRANTE, S. A., ELBASHA, E. H., CHHATWAL, J., ZHANG, X. H. & HSU, T. Y. 2013. Disease progression and cost-effectiveness for chronic hepatitis C virus genotype1: Boceprevir usage with singapore setting. Hepatology International, 7, S432.

D'ANGELO, E. R., GARCIA GONZALEZ, I. & SIMON, M. A. 2012. The use of futility rules in economic evaluations with direct acting agents (daa) in the treatment of genotype 1 hepatitis c virus (HCV) from a spanish health care perspective. Value in Health, 15, A392-A393.

DE LEDINGHEN, V., ORSINI, M., FOUCHER, J., VERGNIOL, J., CHERMAK, F., MESSAOUDI, N. & DAURES, J. P. 2012. Cost-effectiveness of HCV treatment in genotype 1 naive patients in 2012. Journal of Hepatology, 56, S384-S385.

DENIZ, B., BROGAN, A., MILLER, J., TALBIRD, S. & THOMPSON, J. 2011. Cost-effectiveness assessment of telaprevir combination treatment compared to pegylated-interferon+ribavirin alone in the management of hepatitis c in patients who failed prior pegylated-interferon+ribavirin treatment 2011 acg presidential poster. American Journal of Gastroenterology, 106, S410.

EKSTROM, V. S. M., YEE, M. L., CHOW, W. C. & KUMAR, R. 2015. Cost effectiveness of pegylated inteferon and ribavirin combination in treating genotype 1 hepatitis C patients in Singapore. Hepatology International, 9, S268.

JALUNDHWALA, Y. J., MANZOOR, B. S., PATEL, H., CHENG, W. H., PATEL, P. & TOUCHETTE, D. R. 2014. Cost-effectiveness of pharmacotherapies for treatment naive hepatitis C genotype 1 patients: A payer's perspective. Value in Health, 17, A273.

JALUNDHWALA, Y. J., PATEL, P., NANCY, H., WENDY, C., BEENISH, M., HARIDARSHAN, P. & TOUCHETTE, D. 2012. Evaluating the cost-effectiveness of using boceprevir and telaprevir in the treatment of newly diagnosed Hepatitis C genotype 1 patients: A payer's perspective. Pharmacotherapy, 32, e300-e301.

KANG, J., BARON, K., POLHAMUS, D., FRENCH, J. L. & GASTONGUAY, M. R. 2013. Model-based meta-analysis for virologic response rate in hepatitis C virus (HCV) clinical trials. Clinical Pharmacology and Therapeutics, 93, S5.

KHAIRY, A. M., AWAD, A., EL-AKEL, W., DOSS, W., ZAYED, N. & MABROUK, M. 2012. Using data mining techniques to predict sustained virological response in Egyptian patients with chronic Hepatitis C Virus. Journal of Gastroenterology and Hepatology, 27, 251-252.

KUROSAKI, M., SAKAMOTO, N., IWASAKI, M., SAKAMOTO, M., SUZUKI, Y., HIRAMATSU, N., SUGAUCHI, F., SATO, M., TSUCHIYA, K., ASAHINA, Y. & IZUMI, N. 2010. Impact of mutations in ISDR and core region of HCV on pretreatment prediction of sustained virological response to pegylated-interferon plus ribavirin therapy revealed by classification and regression tree analysis. Hepatology International, 4, 190-191.

LA TORRE, G., MIELE, L., MANNOCCI, A., SAULLE, R., GIRALDI, G., UNIM, B., URSILLO, P., SEMYONOV, L., COLAMESTA, V., MELCARNE, R., BIOLATO, M., CECCHI, R., VILLARI, P. & DE GIUSTI, M. 2013. Organizational and economic issues related to the introduction of boceprevir in the treatment of patients with genotype1 chronic hepatitisc in Italy. Value in Health, 16, A494-A495.

LAI, M. N., CHAN, K., GROESSL, E. J. & HO, S. B. 2013. Long term clinical impacts of interferon-free direct antiviral agent therapy for chronic hepatitis C genotype 1 infection in the Veterans Health Administration. Hepatology, 58, 1165A.

LECHUGA, D. & ALVA, M. 2012. Economic evaluation of the use of peg-interferon alfa 2a in the treatment of patients with chronic hepatitis C public Mexican perspective. Value in Health, 15, A136-A137.

LION, M., MCCANN, E. & JIANG, Y. 2013. Cost-effectiveness of peginterferon alfa and ribavirin for the treatment of children and young people with chronic hepatitisc from the perspective of the nhs in England and Wales. Value in Health, 16, A352.

LUKAC, M., BIELIK, J., HOLOMAN, J., TOMEK, D., SUVADOVA, A., FOLTANOVA, T. & FOLTAN, V. 2012. Cost-utility analysis of telaprevir in combination with peginterferon alpha and ribavirin in previously treated patients with chronic hepatitis c. Value in Health, 15, A330.

MACIOCH, T., PAWESKA, J., NIEWADA, M., BERAK, H., SZKULTECKA-DEBEK, M. & RUSSEL-SZYMCZYK, M. 2011. Cost-effectiveness analysis of pegylated interferon alpha-2A versus pegylated interferon alpha-2b in the treatment of chronic hepatitis C patients in Poland. Value in Health, 14, A274-A275.

MAO, W. H., CHEN, W. & WEI, L. 2014. Economic evaluation of viral load test (VLT) in response guided treatment (RGT) for chronic hepatitis C (CHC). Value in Health, 17, A749-A750.

MARCELLUSI, A., VITI, R., CAPONE, A. & MENNINI, F. S. 2014. Direct and indirect cost of HCV-related diseases in Italy: An incidence-based probabilistic approach. Value in Health, 17, A671.

MCDERMOTT, C. L., VEENSTRA, D. L., HANSEN, R. N. & SULLIVAN, S. D. 2013. The value of improving treatment adherence in chronic hepatitis c infection. Value in Health, 16, A214.

MCEWAN, P., WARD, T., KIM, R., L'ITALIEN, G. & YUAN, Y. 2013. Validation of the monarch hepatitis C model to Japanese hepatocellular carcinoma incidence data. Hepatology International, 7, S730-S731.

MCEWAN, P., WARD, T., YUAN, Y. & L'ITALIEN, G. 2013. Assessing the importance of fibrosis stage on the cost-effectiveness of birth-cohort versus risk-based screening and treatment for hepatitis c virus infection. Value in Health, 16, A186.

MCEWAN, P., YUAN, Y. & KIM, R. 2010. A need for a dynamic approach to modelling disease progression in cost-effectiveness studies of antiviral therapies in patients with Chronic Hepatitis C. Hepatology International, 4, 197.

MCEWAN, P., YUAN, Y. & KIM, R. 2012. Evaluating optimal treatment outcomes of antiviral therapy in Hepatitis C for prior null responders in the era of first generation protease inhibitors. Journal of Hepatology, 56, S387.

MCEWAN, P., YUAN, Y., LITALIEN, G. & KIM, R. 2011. Cost benefit analysis of response guided therapy: Dynamic disease Markov modeling for patients with chronic hepatitis (HCV) by fibrosis stages. Journal of Hepatology, 54, S461.

MCEWAN, P., YUAN, Y., TOWNSEND, R. & KIM, R. W. 2010. The impact of age dependent utility on the cost effectiveness of pegylated interferon and ribavirin versus interferon and ribavirin as therapy for genotype 1 patients with chronic hepatitis C. Value in Health, 13, A193.

MCGHAN, A. A. & KAPLAN, D. E. 2012. Costs of palliative treatments of intermediate and advanced stage hepatocellular carcinoma may exceed usually accepted limits of cost effectiveness. Hepatology, 56, 1097A.

MCGINNIS, J. J. & HAY, J. W. 2014. The cost-effectiveness of hepatitis C treatments in treatment naive genotype 1 patients. Value in Health, 17, A274-A275.

MERNAGH, P., FENG, J. & DEL CUORE, M. 2013. Cost-effectiveness of boceprevir therapy in adult patients with chronic hepatitis C (HCV) genotype 1. Value in Health, 16, A94.

MERNAGH, P., FENG, J. & DEL CUORE, M. 2013. Cost-effectiveness of boceprevir therapy in previously treated adult patients with chronic hepatitis C (HCV) genotype 1. Hepatology International, 7, S392.

MERNAGH, P., FENG, J. & DEL CUORE, M. 2013. Cost-effectiveness of boceprevir therapy in untreated adult patients with chronic hepatitis C (HCV) genotype 1. Hepatology International, 7, S391.

MERNAGH, P., NORRIS, S. & DEL CUORE, M. 2011. Anti-viral treatment of chronic hepatitis C in a paediatric population: A cost-effectiveness analysis. Value in Health, 14, A403.

MORAIS, A. D. & PEREIRA, M. L. 2013. Cost-effectiveness of telaprevir plus peginterferon/riba virin (TVR+PR) versus peginterferon/riba virin (PR) in treatment-naive genotype 1 chronic hepatitisc patients with f2 fibrosis in Brazil. Value in Health, 16, A354.

MOURAD, A., DEUFFIC-BURBAN, S., RENAULT-VANTROYS, T., GANNE-CARRIE, N., ROSA, I., BOUVIER, A. M., LAUNOY, G., CATTAN, S., LOUVET, A., DHARANCY, S., TRINCHET, J. C., YAZDANPANAH, Y. & MATHURIN, P. 2013. Ultrasonographic (US) screening of hepatocellular carcinoma (HCC) in compensated cirrhosis due to hepatitis C virus (HCV) improves survival: A modeling approach. Journal of Hepatology, 58, S269.

NIKOGLOU, E., HUMPHREYS, S., EL KHOURY, A., FERRANTE, S. A. & O'REGAN, C. 2011. The clinical efficacy and cost-effectiveness of boceprevir in combination with pegylated interferon-alfa and ribavirin for the treatment of genotype 1 chronic hepatitis C patients: A within trial analysis from the perspective of the scottish national health service (NHS). Value in Health, 14, A274.

NORRIS, S., CAMPBELL, S., RADALJ, L., ELLIOTT, L. & DUNLOP, S. L. 2009. Cost effectiveness of pegylated-interferon alpha 2B + ribavirin for chronic hepatiitis C (CHC) In patients who have previously failed treatment with interferon-based therapy in Australia. Value in Health, 12, A424.

NORTHUP, P. G., AL-OSAIMI, A. M., CALDWELL, S. H. & ARGO, C. K. 2009. Cost effectiveness of STAT-C agents in treating genotype 1 chronic hepatitis C. Hepatology, 50, 671A.

NOVAK, A., DRENTH, J. P. H. & DE KNEGT, R. J. 2014. Costs per successfully treated patient with sofosbuvir in GT1 HCV. Value in Health, 17, A673.

NOVAK, A., DRENTH, J. P. H. & DE KNEGT, R. J. 2014. The pan-genotypic costs-effectiveness of sofosbuvir in hepatitis c virus. Value in Health, 17, A676.

OBACH, D., YAZDANPANAH, Y., ESMAT, G. E., CANVA-DELCAMBRE, V., DEWEDAR, S., ANWAR, W. A., DOSS, W. H., MOSTAFA, A., POL, S., BUTI, M., SIEBERT, U., FONTANET, A., MOHAMED, M. K. & DEUFFIC-BURBAN, S. 2012. Impact of different treatment scale-up and eligibility scenarios on HCV mortality in egypt in the next five years. Hepatology, 56, 1012A.

ODHIAMBO, R., CHHATWAL, J., FERRANTE, S. A., EL KHOURY, A. & ELBASHA, E. 2012. Economic evaluation of boceprevir for the treatment of patients with genotype 1 chronic hepatitis C virus infection in hungary. Value in Health, 15, A390.

OMELYANOVSKY, V. V., AVKSENTIEVA, M. V., KRYSANOV, I. & IVAKHNENKO, O. 2010. Cost-effectiveness analysis of peginterferon (ALFA-2B) with ribavirin compared with peginterferon (ALFA-2A) with ribavirin for the treatment of chronic hepatitis c. Value in Health, 13, A431.

OZDEMIR, O. 2014. The cost-effectiveness of telaprevir triple therapy in treatment of naive chronic hepatitis c patients in Turkey. Value in Health, 17, A670.

PAPATHEODORIDIS, G. V. & ARZOUMANIDOU, D. 2012. Cost-effectiveness analysis of different protease inhibitors-based strategies in the treatment of naive patients with genotype 1 chronic hepatitis C (G1-CHC). Hepatology, 56, 1014A.

PAREKH, H., MCGARRY, L., PAWAR, V., DENIZ, B. & WEINSTEIN, M. 2011. Projected lifetime economic burden of hepatitis C virus in U.S. Birth cohorts with high prevalence. Journal of Managed Care Pharmacy, 17, 567-568.

PARRONDO, J., RINCON RODRIGUEZ, D., ROMERO GOMEZ, M., SOLA LAMOGLIA, R. & ROLDAN, C. 2014. Cost-effectiveness analysis of eltrombopag as support treatment in chronic HCV infected patients with thrombocytopenia to enable interferon-based regimens. Value in Health, 17, A532.

PAVELIU, M. S., COMSA, R. & MIRCEA, R. 2013. Cost-utility analysis of telaprevir in combination with peginterferon alpha and ribavirin in previously untreated patients with chronic hepatitis in a romanian setting. Value in Health, 16, A499.

PIRATVISUTH, T., PRAMOOLSINSAP, C., CHUTAPUTTI, A., TANWANDEE, T., THONGSAWAT, S., SUKAROM, I., TINMANEE, S. & BERGER, W. 2009. Cost-effectiveness analysis of peginterferon alfa-2a (40 KD) plus ribavirin versus no treatment in patients with chronic hepatitis C (CHC), genotype 1 from a payer perspective in Thailand. Hepatology, 50, 714A-715A.

PYADUSHKINA, E., AVXENTYEVA, M., OMELYANOVSKY, V. V., TREUR, M. & WESTERHOUT, K. Y. 2014. Cost-effectiveness analysis of antiviral pharmac otherapies for treatment of chronic hepatitis C virus infection in Russia. Value in Health, 17, A366-A367.

RAMACHANDRAN, S., MAHABALESHWARKAR, R. & YANG, Y. 2012. Cost effectiveness analysis of addition of telaprevir or boceprevir to standard therapy versus standard therapy alone for the treatment of previously untreated chronic Hepatitis-C virus genotype 1 infection. Value in Health, 15, A242.

REIN, D. B. 2013. A review and summary analysis of major studies of the cost-effectiveness birth cohort testing for hepatitis c in United States. Hepatology, 58, 391A.

REIN, D. B., WITTENBORN, J. S., LIFFMANN, D. K., HASCHE, J. C., BORTON, J. M., KRAMER, J. R., DUAN, Z. & EL-SERAG, H. B. 2014. Projected health and economic impact of hepatitis c on the united states veterans administration health system from 2014 to 2024. Journal of Hepatology, 60, S332.

RUGGERI, M. 2014. Using bayesian modelling to estimate the cost effectiveness of telaprevir and boceprevir in Italian naive patients with hepatitis C. Value in Health, 17, A277.

RYAZHENOV, V. V., SBOYEVA, S. G. & EMCHENKO, I. V. 2013. Pharmac oeconomic analysis of different antiviral therapies in the treatment of Russian patients with chronic hepatitisc. Value in Health, 16, A345.

SAAB, S., GORDON, S. C., PARK, H., AHMED, A. & YOUNOSSI, Z. M. 2014. A decision analytic markov model to evaluate the health outcomes of sofosbuvir for previously untreated patients and those without treatment options with chronic hepatitis C virus. Gastroenterology, 146, S-912.

SAID, M., DRAGOSITS, A. & WALTER, E. 2012. Cost-effectiveness-analysis of the combination-therapy telaprevir, peg-ifn+2a and ribavirin in patients with chronic hepatitis-C in austria. Value in Health, 15, A392.

SALINAS ESCUDERO, G., IDROVO, J., RIVAS, R., RAMIREZ RODRIGUEZ, J., RICO ALBA, I. A. & ZAPATA, L. 2009. Cost-effectiveness of peg-ifn alpha 2A OR 2B plus ribavirin in the treatment of chronic hepatitis C in Mexico. Value in Health, 12, A59.

SALINAS ESCUDERO, G., IDROVO, J., RIVAS, R., RAMIREZ RODRIGUEZ, J., RICO ALBA, I. A. & ZAPATA, L. 2009. Cost-effectiveness of PEG-IFN alpha 2A OR 2B plus ribavirin in the treatment of chronic hepatitis C in Mexico. Value in Health, 12, A508.

SIEBERT, U., MUHLBERGER, N., CONRADS-FRANCENK, A., SROCZYNSKI, G. & SCHWARZER, R. 2009. Using Iqwig's efficiency frontier approh for the economic evaluation of heaptitis c treatment - a pilot and feasibility study commissioned by IQWIG. Value in Health, 12, A225.

SILVA, M., FELIX, J., FERREIRA, D., VANDEWALLE, B., GUERRA, I., CURE, S., ALDIR, I., CARVALHO, A., MACEDO, G., MARINHO, R. T., PEDROTO, I. & RAMALHO, F. 2014. Sofosbuvir for the treatment of chronic hepatitis C: A comprehensive cost-effectiveness analysis across hcv genotypes, pretreatment conditions and HIV co-infection. Value in Health, 17, A366.

STAHMEYER, J. T., SCHAUER, S., WIRTH, D., FLEISCHMANN, J., LEE, S., GAVART, S., BIANIC, F., CURE, S. & KRAUTH, C. 2012. Cost-effectiveness of triple therapy with telaprevir for the treatment of treatment-naive genotype 1 chronic hepatitis c patients in germany. Value in Health, 15, A396.

SYPSA, V., MAGIORKINIS, G., MAGIORKINIS, E., PARASKEVIS, D., KATSOULIDOU, A. S., PAPATHEODORIDIS, G. V. & HATZAKIS, A. 2012. The impact of primary prevention and treatment as prevention in controlling the epidemic of hepatitis C under the new therapeutic options. Hepatology, 56, 1051A.

TAN, S. S., FERRANTE, S. A., ELBASHA, E. H., CHHATWAL, J., OMAR, H., KIEW, K. K., HASSAN, M. R. A., MENON, J. & HSU, T. Y. 2013. The cost-effectiveness of boceprevir based triple therapy for genotype 1 hepatitis C in the settings of malaysia's public sector healthcare. Hepatology International, 7, S723-S724.

THONGSAWAT, S., PIRATVISUTH, T., PRAMOOLSINSAP, C., CHUTAPUTTI, A., TANWANDEE, T., BERGER, W., SUKAROM, I. & TINMANEE, S. 2009. Peginterferon alfa-2a (40kd) plus ribavirin is dominant with large cost-savings versus no treatment in patients with chronic hepatitis C (CHC), genotype 2/3: A cost-effectiveness analysis from the Thai payer perspective. Hepatology, 50, 704A-705A.

TSOCHATZIS, E., CROSSAN, C., LONGWORTH, L., GURUSAMY, K., RODRIGUEZ-PERALVAREZ, M., MANTZOUKIS, K., O'BRIEN, J., THALASSINOS, E., PAPASTERGIOU, V., NOEL-STORR, A., DAVIDSON, B. & BURROUGHS, A. 2014. Cost-effectiveness of non-invasive liver fibrosis tests for treatment decisions in patients with chronic hepatitis c: Systematic review and economic evaluation. Journal of Hepatology, 60, S33.

VELLOPOULOU, K., VAN AGTHOVEN, M., VAN DER KOLK, A., LAMOTTE, M., CURE, S. & BIANIC, F. 2012. The cost-utility of telaprevir in combination with peginterfeon alpha and ribavirin (pr) as compared to the combination boceprevir with pr and to pr alone in the management of chronic hepatitis c in the netherlands. Value in Health, 15, A396.

VENTAYOL-BOSCH, P., RUBIO-TERRES, C., GARCIA-SAMANIEGO, J., PLANAS, R., SOLA-LAMOGLIA, R., ROMERO-GOMEZ, M., DIAGO-MADRID, M., CRESPO-GARCIA, J., CALLEJA-PANERO, J. L. & TURNES-VAZQUEZ, J. 2010. Cost-effectiveness analysis of treatment of chronic hepatitis c patients with peginterferon alfa-2a or peginterferon alfa-2b both plus ribavirin in Spain. Value in Health, 13, A438.

WANG, Y. D., CHEN, G. F., WU, V., WONG, A., CHAU, A., SHAO, Q., LI, F., LI, B., JI, D., LI, Z., CHEN, S. H., WANG, C. Y., NIU, X. X., DING, S., YAN, T. & LAU, G. K. K. 2015. Cost effectiveness of sofosbuvir based therapy for chronic HCV genotype 1b infection in China. Hepatology International, 9, S53.

WANG, Y. D., CHEN, G. F., WU, V., WONG, A., CHAU, A., SHAO, Q., LI, F., LI, B., JI, D., LI, Z., CHEN, S. H., WANG, C. Y., NIU, X. X., DING, S. Y., YAN, T. & LAU, G. K. K. 2015. Cost effectiveness of sofosbuvir based therapy for chronic HCV genotype 1b infection in China. Hepatology International, 9, S47.

WARD, T., WILTON, H. B., MCEWAN, P., KALSEKAR, A. & YUAN, Y. 2014. Assessing the health economic value of each unit of SVR improvement in HCV infection. Hepatology, 60, 1047A.

WESTERHOUT, K. Y., TREUR, M. & CERRI, K. 2013. Effect of antiviral treatment rates on the predicted future burden of genotype-1 chronic hepatitisc in the United Kingdom. Value in Health, 16, A342.

WONG, J. B., THORNTON, K. A., CARROLL, C. & ARORA, S. 2013. Cost-effectiveness of hepatitis c treatment by primary care providers supported by the Extension for Community Healthcare Outcomes (ECHO) Model. Hepatology, 58, 330A.

WOODS, M. S., KIRI, S., LING, C., MCCRINK, L., ZIMOVETZ, E. & HASS, B. 2013. A systematic review of economic evidence in hepatitisc: An ov erview of cost, utility and cost-effectiveness data. Value in Health, 16, A349.

WOODS, M. S., KIRI, S., LING, C., MCCRINK, L., ZIMOVETZ, E. & HASS, B. 2013. A systematic review of economic evidence in hepatitisc: Methods used in recent economic evaluations. Value in Health, 16, A579.

YFANTOPOULOS, J., PAPAROUNI, K. & D'ANGELO, E. R. 2012. A cost-effectiveness analysis of telaprevir versus boceprevir in the treatment of hepatitis C: A greek national health system perspective. Value in Health, 15, A394.

YOUNOSSI, Z., GORDON, S., SAAB, S., AHMED, A., PARK, H. & SULKOWSKI, M. 2014. Health and economic outcomes of sofosbuvir therapy as predicted by a markov model in the HCV/HIV co-infected cohort. Value in Health, 17, A37.

YOUNOSSI, Z., SAAB, S., AHMED, A. & GORDON, S. C. 2014. A decision-analytic markov model to evaluate the health outcomes of ledipasvir/sofosbuvir (LDV/SOF) for patients with chronic hepatitis c (HCV) genotype 1 (GT1) infection. Hepatology, 60, 1043A.

YOUNOSSI, Z., SINGER, M., HENRY, L., HUNT, S. L., JEFFERS, T., FROST, S. & LAM, B. P. 2014. The use of all oral regimens for treatment of chronic hepatitis C (CHC) coupled with birth cohort screening is highly cost effective: The health and economic impact on the U.S. population. Hepatology, 60, 256A.

YOUNOSSI, Z. M., GORDON, S., SAAB, S., AHMED, A., CURE, S. & GUERRA, I. 2013. A decision analytic markov model to evaluate the health outcomes of sofosbuvir for previously untreated patients and those without treatment options with chronic hepatitisc virus genotype 2 infection. Value in Health, 16, A341.

YOUNOSSI, Z. M., GORDON, S. C., SAAB, S., AHMED, A., BROGAN, A. & CURE, S. 2013. A decision analytic Markov model to evaluate the health outcomes of sofosbuvir for previously untreated patients with chronic hepatitis C virus genotype 1 infection. Hepatology, 58, 387A-388A.

YOUNOSSI, Z. M., SAAB, S., GORDON, S. C., AHMED, A., BROGAN, A. & GUERRA, I. 2013. Evaluation of the long-term health outcomes associated with earlier versus later initiation of treatment in previously untreated patients with chronic hepatitis C virus genotype 1 infection. Hepatology, 58, 388A.

YOUNOSSI, Z. M., SINGER, M. & MIR, H. M. 2013. The potential impact of interferon free oral regimens on clinical and cost outcomes of patients with chronic hepatitis C, genotype 1: A decision analytic assessment. Journal of Hepatology, 58, S210.

YOUNOSSI, Z. M., SINGER, M. E. & MIR, H. M. 2013. Interferon-based and interferon-free regimens for patients with chronic hepatitis c, genotype 1: Potential cost-effectiveness of biopsy-guided treatment versus treat all strategies. Gastroenterology, 144, S988.

ZHOU, K., FERGUSON, J., ELASHOFF, D. & SAAB, S. 2014. Treating chronic hepatitis C infection in the elderly: Estimated impact on life expectancy. Gastroenterology, 146, S-969.

**Reason for exclusion, language (n=14)**

BUTI, M. & CASADO, M. A. 2003. Cost-benefit analysis of combined therapy in chronic hepatitis C. Enfermedades Emergentes, 5, 90-96.

BUTI, M., CASADO, M. A., FOSBROOK, L. & ESTEBAN, E. 1998. [Cost effectiveness of the treatment of chronic hepatitis C with interferon-alpha]. 21, 161-168.

BUTI, M., CASADO, M. A., FOSBROOK, L. & ESTEBAN, R. 1998. [Cost effectiveness of the treatment of chronic hepatitis C with interferon-alpha]. Gastroenterol Hepatol, 21, 161-8.

BUTI, M., GROS, B., OYAGUEZ, I., ANDRADE, R. J., SERRA, M. A., TURNES, J. & CASADO, M. A. 2014. [Cost-utility analysis of triple therapy with telaprevir in treatment-naive hepatitis C patients]. 38.

CASADO GOMEZ, M. A. 2006. Cost-effectiveness of pegylated interferon-alfa in the treatment of chronic hepatitis due to HCV. Gastroenterologia Hepatologia, 29, 200-205.

DE MELLO VIANNA, C. M., BITTENCOURT GONZALEZ MOSEGUI, G., COSTA E SILVA, F. V., PEREGRINO, A. A. F., DA SILVA RODRIGUES, M. P. & JARDIM, F. N. 2013. Avaliação Econômica do Interferon Peguilado Alfa 2a em Combinação com a Ribavirina para o Tratamento da Infecção Pelo Genótipo 1 da Hepatite Crônica C: Comparação do Tratamento por 48 Semanas e Terapia Estendida. Value in Health Regional Issues 2, 342-346.

FERNANDES, F. F. 2008. Prognosis and associated factors to the effectiveness of chronic hepatitis c treatment.

GARCIA-JURADO, L., OYAGUEZ, I., CASADO, M. A., TURAL, C., GONZALEZ-GARCIA, J., ORTEGA, E. & PINEDA, J. A. 2012. [Evaluation of the costs of transient elastography (FibroScan((R))) in the diagnosis of liver fibrosis in HIV patients with hepatitis C virus]. Enferm Infecc Microbiol Clin, 30, 294-9.

HAJ-ALI SAFLO, O. & HERNANDEZ GUIJO, J. M. 2009. Cost-effectiveness of chronic hepatitis C treatment in Spain. Gastroenterologia y Hepatologia, 32, 472-482.

HUZICKA, I. & BIELIK, J. 2006. Economic effectivity of chronic hepatitis C (CHC) treatment by peginterferon alpha-2b and ribavarin - Slovak farmacoeconomy study. Ceska e Slovenska Gastroenterologie a Hepatologie, 60, 83-89.

IUSHCHUK, N. D., ZNOIKO, O. O., IAKUSHECHKINA, N. A., ZYRIANOV, S. K., SHUT'KO, S. A., BELYI, P. A., KOZINA, A. N., CHAPURIN, S. A., CHURILIN, I. I. & LOUGOVSKIKH, E. A. 2013. [The burden of viral hepatitis in the Russian Federation requires reducing its progression for the long term, including chronic hepatitis C]. Terapevticheskii Arkhiv, 65, 79-85.

WASEM, J., SROCZYNSKI, G., AIDELSBURGER, P., BUCHBERGER, B., HESSEL, F., CONRADS-FRANK, A., PETERS-BLOCHINGER, A., KURTH, B. M., WONG, J. B., ROSSOL, S. & SIEBERT, U. 2006. [Health economics of chronic infectious diseases: the example of hepatitis C]. Bundesgesundheitsblatt Gesundheitsforschung Gesundheitsschutz, 49, 57-63.

YOSHIDA, H., TATEISHI, R. & OMATA, M. 2004. [Cost-effectiveness of antiviral therapy for chronic hepatitis C patients in prevention of hepatocellular cancer ]. Nihon Rinsho, 62 Suppl 7, 511-4.

ZECHMEISTER, I. & WILD, C. 2006. Chronic hepatitis C: decision analytic modelling (structured abstract). Vienna (Austria): Institute of Technology Assessment (ITA). ITA-Projektbericht 29. 2006.

**Reason for exclusion, review (n=1)**

TOWNSEND, R., MCEWAN, P., KIM, R. & YUAN, Y. 2011. Structural frameworks and key model parameters in cost-effectiveness analyses for current and future treatments of chronic hepatitis C. Value Health, 14, 1068-77.

**Reason for exclusion, duplicate (n=1)**

SHEPHERD, J., JONES, J., HARTWELL, D., DAVIDSON, P., PRICE, A. & WAUGH, N. 2007. Interferon alpha (pegylated and non-pegylated) and ribavirin for the treatment of mild chronic hepatitis C: a systematic review and economic evaluation. Health Technol Assess, 11, 1-205, iii.

**Reason for exclusion, unobtainable (n=2)**

DORE, G. J., FREEMAN, A. J., LAW, M. & KALDOR, J. M. 2003. Natural history models for hepatitis C-related liver disease: different disease progression parameters for different settings. Antivir Ther, 8, 365-72.

KIM, D. D., HUTTON, D. W., RAOUF, A. A., SALAMA, M., HABLAS, A., SEIFELDIN, I. A. & SOLIMAN, A. S. 2012. Cost effectiveness of implementing a screening and treatment program for hepatitis C virus infection in Egypt. Cancer Research, 72.

**Reason for exclusion, no usable data (n=2)**

ARGUEDAS, M. 2004. Cost effectiveness of peginterferon alpha-2a plus ribavirin versus interferon alpha-2b plus ribavirin as initial therapy for treatment-naive chronic hepatitis C. Pharmacoeconomics, 22, 477-8; author reply 478-9.

YOUNOSSI, Z. M., HENRY, L. & SINGER, M. 2014. Reply to: "Cost-effectiveness of upcoming treatments for hepatitis C: we need to get the models right". J Hepatol, 61, 454-5.
